# Disulfiram Overcomes Cisplatin Resistance in Human Embryonal Carcinoma Cells

**DOI:** 10.3390/cancers11091224

**Published:** 2019-08-22

**Authors:** Silvia Schmidtova, Katarina Kalavska, Katarina Gercakova, Zuzana Cierna, Svetlana Miklikova, Bozena Smolkova, Verona Buocikova, Viera Miskovska, Erika Durinikova, Monika Burikova, Michal Chovanec, Miroslava Matuskova, Michal Mego, Lucia Kucerova

**Affiliations:** 1 Cancer Research Institute, Biomedical Research Center, University Science Park for Biomedicine, Slovak Academy of Sciences, Dubravska cesta 9, 845 05 Bratislava, Slovakia; 2 Department of Oncology, Faculty of Medicine, Comenius University and National Cancer Institute, Klenova 1, 833 10 Bratislava, Slovakia; 3 Translational Research Unit, Faculty of Medicine, Comenius University, Klenova 1, 833 10 Bratislava, Slovakia; 4 Department of Pathology, Faculty of Medicine, Comenius University, Sasinkova 4, 811 08 Bratislava, Slovakia; 5 Department of Oncology, Faculty of Medicine, Comenius University and St. Elisabeth Cancer Institute, Kolarska 12, 812 50 Bratislava, Slovakia

**Keywords:** testicular germ cell tumors, embryonal carcinoma, chemoresistance, cisplatin, cancer stem cell markers

## Abstract

Cisplatin resistance in testicular germ cell tumors (TGCTs) is a clinical challenge. We investigated the underlying mechanisms associated with cancer stem cell (CSC) markers and modalities circumventing the chemoresistance. Chemoresistant models (designated as CisR) of human embryonal carcinoma cell lines NTERA-2 and NCCIT were derived and characterized using flow cytometry, gene expression, functional and protein arrays. Tumorigenicity was determined on immunodeficient mouse model. Disulfiram was used to examine chemosensitization of resistant cells. ALDH1A3 isoform expression was evaluated by immunohistochemistry in 216 patients’ tissue samples. Chemoresistant cells were significantly more resistant to cisplatin, carboplatin and oxaliplatin compared to parental cells. NTERA-2 CisR cells exhibited altered morphology and increased tumorigenicity. High ALDH1A3 expression and increased ALDH activity were detected in both refractory cell lines. Disulfiram in combination with cisplatin showed synergy for NTERA-2 CisR and NCCIT CisR cells and inhibited growth of NTERA-2 CisR xenografts. Significantly higher ALDH1A3 expression was detected in TGCTs patients’ tissue samples compared to normal testicular tissue. We characterized novel clinically relevant model of chemoresistant TGCTs, for the first time identified the ALDH1A3 as a therapeutic target in TGCTs and more importantly, showed that disulfiram represents a viable treatment option for refractory TGCTs.

## 1. Introduction

Testicular germ cell tumors (TGCTs) represent the most common solid tumors among adolescent and young men between the age of 20 to 40 [1]. TGCTs are classified into two histopathological categories—seminoma and non-seminoma, consisting of embryonal carcinoma (EC), teratoma, choriocarcinoma, yolk sac tumor and sometimes seminoma [2]. Among them, EC represents the most common non-seminomatous germ cell tumor (NSGCT) present in ~77% of mixed NSGCTs [3].

To date, almost 80% of patients with TGCTs including the ones with disseminated disease are cured by cisplatin-based chemotherapy [4]. However, 15–30% of the patients with metastatic disease do not achieve a complete remission, have a poor prognosis and succumb to a progressive disease [5,6]. Even though molecular pathways implicated in a resistance to cisplatin-based chemotherapy in TGCTs patients were extensively studied, our knowledge in this field of research is still limited [7,8]. Identifying novel therapeutic targets based on molecular and genetic characteristics of chemoresistant TGCTs could improve treatment outcome, not only in testicular tumors but also in other solid tumors such as ovarian, breast, head and neck, and non-small cell lung cancers, where cisplatin-based chemotherapy remains the first line treatment [9].

Previously, chemoresistance in solid tumors was associated with an upregulation of cancer stem cells markers [10,11]. Increased expression of pluripotency transcription factors Sox2, Oct4, Nanog, Klf4, Hes1, receptor Lgr5, surface marker molecules CD24, CD26, CD44, CD133, CD166, isoforms of aldehyde dehydrogenase (ALDH), and ATP binding cassette subfamily G member 2 (ABCG2) was detected in cisplatin-resistant cell line variants derived from lung adenocarcinoma, malignant pleural mesothelioma and non-small cell lung cancer cells [12,13,14]. Overall ALDH activity, measured by the Aldefluor assay, was also described as one of CSCs markers. Nine ALDH isoforms were identified potentially contributing to ALDH activity and they exhibit different expression patterns in different cancer types. However, mainly ALDH1 family members (ALDH1A1, ALDH1A2, and ALDH1A3) contribute to enhanced self-renewal, survival, and proliferation of CSCs [15]. These markers were not analyzed in the cisplatin-resistant TGCT models so far. Moreover, limited number of such models is available, even though they represent a prerequisite for the analysis of mechanisms included in the treatment resistance. Cisplatin-resistant variants of EC cell lines, namely NTERA-2, NCCIT, 2102EP, seminoma cell line TCam-2, and testicular non-seminomatous germ cell line GCT27 were derived from established cell lines by a long-term exposure of the parental cells to the cisplatin in vitro [16,17,18,19,20]. Cisplatin-resistant human embryonal carcinoma NT2/D1-R1 cells and non-seminoma germ cell tumor H12.1 RA cells were derived by inducing terminal differentiation in these cells by retinoic acid (RA) [21,22]. The cell line H12.1 is cisplatin-sensitive, whereas 1411HP established from a retroperitoneal metastatic tumor resected after chemotherapy is resistant to cisplatin and other conventional chemotherapeutic drugs [22,23].

The aim of our study was to derive novel model for an investigation of the mechanism of cisplatin resistance in TGCTs and its association with upregulation of stemness markers. Moreover, we employed 3D spheroid protocol for the chemosensitivity evaluation as it better predicted tumor responses to the treatment in vivo [24,25]. In our report we characterized novel cisplatin-resistant variant of NTERA-2 and NCCIT germ cell tumor cells designated NTERA-2 CisR and NCCIT CisR with high ALDH activity previously associated with a cancer stem cell phenotype. More importantly, ALDH inhibitor disulfiram restored the sensitivity to cisplatin upon combinatorial treatment in both resistant cell lines and significantly inhibited tumor growth in vivo in NTERA-2 CisR cells. In order to verify the clinical relevance of these findings, we analyzed 216 patient samples and confirmed significant upregulation of the ALDH isoform ALDH1A3 in all histological subtypes of testicular tumors.

## 2. Results

**Figure 1:**
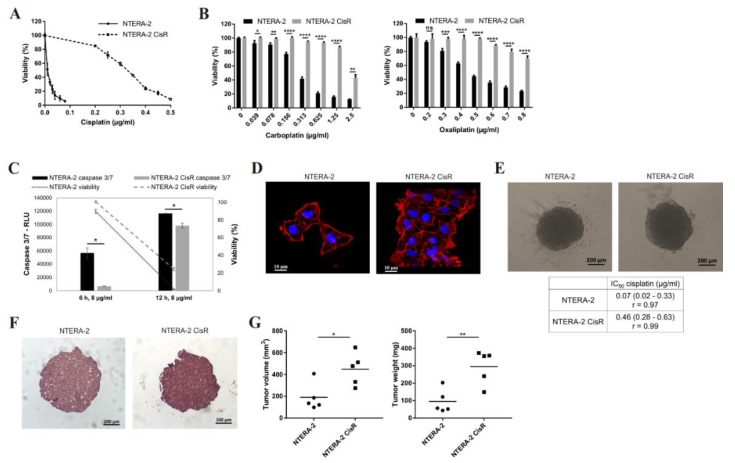
Chemoresistant NTERA-2 CisR cells are cross-resistant to platinum-based drugs, exhibit morphological alterations and increased tumorigenicity. (**A**,**B**) Cytotoxicity of cisplatin, carboplatin and oxaliplatin in NTERA-2 CisR cells was significantly lower in comparison to parental cells. Relative viability was determined by luminescent viability assay on day 6 (carboplatin, oxaliplatin) or 7 (cisplatin). Values were expressed as the averages of quadruplicates ± SD. (**C**) Luminometric measurement of caspase 3/7 activity in 2 timepoints showed decreased activity of this caspase in NTERA-2 CisR cells after cisplatin treatment. Cisplatin sensitivity of resistant cells was lower compare to parental cells. Values were determined as mean values of RLU ± SD and in the case of viability as the averages of quadruplicates ± SD. (**D**) Star-like shape of NTERA-2 CisR cells illustrates alteration in cellular morphology, magnification 630×. (**E**) NTERA-2 CisR cells formed tight 3D multicellular spheroids, when seeded into ultra-low attachment round bottom plates compared to loose spheroids formed by parental NTERA-2 cells, magnification 50×. (**F**) Hematoxylin and eosin staining showed more compact structure of NTERA-2 CisR spheroids, magnification 100×. (**G**) Subcutaneously (s.c.) injected NTERA-2 CisR cells form bigger xenografts then NTERA-2 cells as determined by mean of tumor volume and weight. 2 × 10^5^ of NTERA-2 and NTERA-2 CisR cells were injected s.c. into the flank of immunodeficient mice, and tumor volume and weight were measured. * *p* < 0.05, ** *p* < 0.01, *** *p* < 0.001, **** *p* < 0.0001.

In order to derive a chemoresistant variant from the parental tumor cell line NTERA-2, cells were exposed to gradually increasing concentrations of cisplatin for six months starting from the IC_10_ (0.05 μg/mL); as soon as the cells started to proliferate in the given concentration, it was increased two-fold in the next step. Newly derived NTERA-2 CisR cells were able to grow continuously in 0.1 µg/mL cisplatin at the end of the selection process. The IC_50_ value for cisplatin increased from 0.01 µg/mL in NTERA-2 to 0.31 µg/mL in NTERA-2 CisR, which is 31-fold increase in resistance (Figure 1A). Newly derived cisplatin-resistant NTERA-2 CisR cells were propagated in cisplatin concentrations as stated above and used for further experiments.

NTERA-2 CisR cells were cross-resistant to other platinum-based drugs being 6-fold more resistant to carboplatin and 13-fold more resistant to oxaliplatin (Figure 1B). NTERA-2 CisR cells had significantly decreased levels of activated caspase 3/7 compared to sensitive cells 6 and 12 h post cisplatin treatment. Significantly higher viability was detected in the resistant cells during the treatment at early and later timepoints (Figure 1C).

The immunostaining with an α-F-actin showed that NTERA-2 CisR cells exhibited star-like shape, not seen in the parental cells illustrating alterations in the cellular morphology associated with the development of the chemoresistance (Figure 1D). Changes in the cellular morphology were previously described in various chemoresistant cell line models [26,27,28]. We were able to propagate NTERA-2 and NTERA-2 CisR cells in the 3D non-adherent culture conditions (Figure 1E), which enabled us to determine the chemosensitivity in the 3D conditions. Of note, the chemoresistant NTERA-2 CisR cells formed significantly bigger spheroids (mean spheroid volume: 0.060 ± 0.002 mm^3^ (NTERA-2); 0.077 ± 0.001 mm^3^ (NTERA-2 CisR); *p* < 0.0001). The chemosensitivity in 3D multicellular spheroids was lower compared to the monolayer culture, as expected, and NTERA-2 CisR cells retained significantly higher chemoresistance under these culture conditions (6.6-fold), the IC_50_ values were: IC_50_ (NTERA-2) = 0.07 µg/mL cisplatin; IC_50_ (NTERA-2 CisR) = 0.46 µg/mL cisplatin. Hematoxylin and eosin staining of spheroids showed that NTERA-2 CisR cells formed also more compact spheroids (Figure 1F).

**Figure 2:**
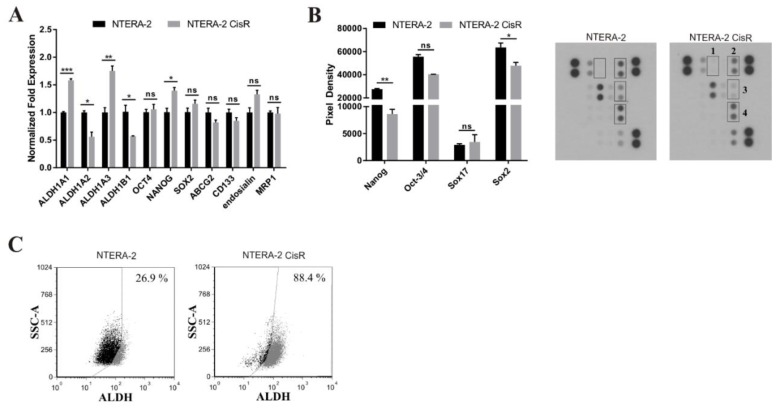
Changes in gene and protein expression of stemness-related markers in cisplatin-resistant cells. (**A**) Expression of ALDH1 isoforms and *NANOG* was significantly changed in NTERA-2 CisR cells as determined by qRT-PCR. (**B**) The cisplatin-resistant NTERA-2 CisR cells exhibited significantly decreased levels of Nanog and Sox2, and non-significant decrease of Oct-3/4. Array spots were visualized in accordance with the manufacturer’s instructions and representative pictures are shown. 1—Sox17, 2—Oct-3/4, 3—Nanog, 4—Sox2. (**C**) Increased ALDH activity was detected in NTERA-2 CisR cells by the Aldefluor assay. The gate for ALDH+ cells was determined in relation to the DEAB control and showed the brightly fluorescent ALDH population versus the side scatter. This population was absent/decreased in the presence of DEAB. The number shown in each panel determined the percentage of ALDH+ cells. HT-29/EGFP/FUR were used for the assay setup as a positive control. * *p* < 0.05, ** *p* < 0.01, *** *p* < 0.001.

As a next step, the tumorigenicity of NTERA-2 CisR cells was examined in SCID mice (Figure 1G). Mean of tumor volume in parental NTERA-2 group was 190 mm^3^ in contrast to NTERA-2 CisR-derived tumor xenografts (mean 449 mm^3^) being almost 60% lower in comparison to the resistant cell line by day 22. The mean of tumor weight in NTERA-2 CisR group was 3-times higher in contrast to NTERA-2 group (295 mg vs. 96 mg). Migratory capacity was analyzed in the 3D spheroid migration assay (Figure S1A). Multivariate analysis of repeated measures showed no differences in migratory capacity between NTERA-2 and NTERA-2 CisR spheroids after 24 h (Figure S1B). NTERA-2 CisR spheroids were still compact after 96 h post placing on the top of conventional culture plates, whereas NTERA-2 spheroids disintegrated (Figure S1C). Gene expression alterations in the genes associated with stemness such as aldehyde dehydrogenase 1—ALDH1 isoforms (*ALDH1A1, ALDH1A2, ALDH1A3, ALDH1B1*), octamer-binding transcription factor 4 (*OCT4*), Nanog homeobox (*NANOG*), sex determining region Y-box 2 (*SOX2*), ATP binding cassette subfamily G member 2 (*ABCG2*), prominin-1 (*CD133*), endosialin—microvascular marker implicated in tumor angiogenesis, and multidrug resistance-associated protein 1 (*MRP1*) were assessed (Figure 2A). The analysis showed significant upregulation of the *ALDH1A1*, *ALDH1A3*, and *NANOG*, and downregulation of *ALDH1A2* and *ALDH1B1* genes in NTERA-2 CisR cells. Representative agarose gel electrophoresis of quantitative real-time PCR (qPCR) amplicons including positive controls is shown in Figure S2.

In order to further analyze properties of the chemoresistant cells, we compared protein expression of selected stemness markers such as Nanog, Oct-3/4, Sox17 and Sox2 in parental and resistant NTERA-2 cell lines. Nanog and Sox2 proteins were significantly decreased in NTERA-2 CisR cells. The expression of Oct-3/4 was decreased too, but this decrease was not significant (Figure 2B). Overall ALDH activity was increased in NTERA-2 CisR compared to NTERA-2 cells, as confirmed by the flow cytometry (Figure 2C). 

**Figure 3:**
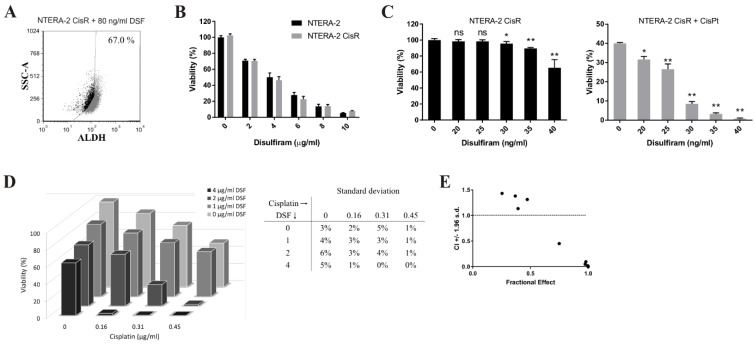
Disulfiram sensitizes chemoresistant NTERA-2 CisR cells to cisplatin. (**A**) Disulfiram alone decreased activity of ALDH for more than 20% in NTERA-2 CisR cells compared to untreated cells in the Aldefluor assay. (**B**) Relative viability of NTERA-2 CisR 3D spheroids after 6 days of disulfiram treatment did not change significantly compared to NTERA-2 cells. Disulfiram exhibited its cytotoxicity in a dose-dependent manner. (**C**) Viability of NTERA-2 CisR cells after 7 days combined treatment with disulfiram and 0.3 µg/mL cisplatin was significantly lower in adherent culture conditions. (**D**) Combined treatment with cisplatin and disulfiram for 6 days very efficiently inhibited propagation and eradicated NTERA-2 CisR 3D spheroids. SD are indicated in the table. (**E**) Data obtained by luminometric assay were subsequently analyzed by Calcusyn software, and Fa-CI plots were created—CI on the y-axis is a function of effect level (fraction affected, Fa) on the x-axis (Fa = 1—% of viable cells/100). Plots display synergism (CI < 1), additivity (CI = 1) or antagonism (CI > 1) for the entire spectrum of effects [29]. Relative viability was determined by luminescent viability assay. Values were expressed as the averages of quadruplicates ± SD. * *p* < 0.05, ** *p* < 0.01.

To investigate whether the resistance could be reversed by the ALDH inhibition, the resistant cells were treated with disulfiram, the inhibitor of the ALDH, in combination with cisplatin. Disulfiram alone decreased ALDH activity by more than 20% in NTERA-2 CisR after 24 h (Figure 2C and Figure 3A). This ALDH inhibitor exhibited dose-depend cytotoxicity on NTERA-2 and NTERA-2 CisR multicellular spheroids and maintained equal cytotoxic effect on both cell lines (Figure 3B). Combined treatment by 30 ng/mL disulfiram and 0.3 µg/mL cisplatin significantly inhibited cell proliferation. Cisplatin treatment alone decreased cell viability by 60% in contrast to the combinatorial treatment, where there was more than 90% inhibition of cell proliferation (Figure 3C). The combination of cisplatin and disulfiram had strong synergistic effect in the 3D culture conditions, where the cisplatin cytotoxicity was augmented after exposure to the disulfiram. Viability of NTERA-2 CisR spheroids decreased by 15% upon treatment with 0.16 μg/mL cisplatin, however, 4 μg/mL disulfiram with cisplatin completely eradicated tumor cells and achieved almost 100% decrease in tumor cell viability (Figure 3D). The combination index (CI) was close to zero indicating strong synergy of higher concentrations of the disulfiram and cisplatin (Figure 3E).

**Figure 4:**
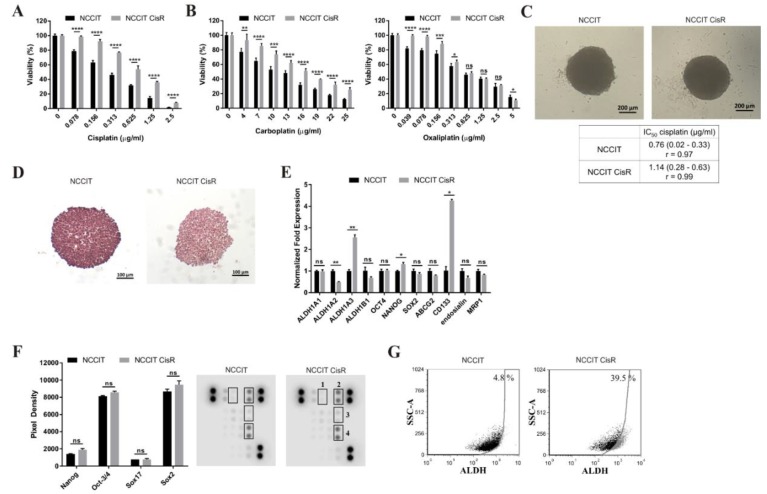
Cisplatin-resistant NCCIT CisR cells differ in sensitivity to platinum-based drugs and exhibit alterations in the expression of genes and proteins associated with stemness. (**A**,**B**) Cytotoxic effect of cisplatin, carboplatin and oxaliplatin in NCCIT CisR cell line was significantly lower compared to NCCIT cells. Relative viability was determined by luminescent viability assay on day 3. Values were expressed as the averages of quadruplicates ± SD. (**C**) NCCIT and NCCIT CisR cells are able of 3D multicellular spheroid formation, when seeded into ultra-low attachment round bottom plates, magnification 50×. (**D**) Hematoxylin and eosin staining showed no differences in structure of NCCIT and NCCIT CisR spheroids, magnification 100×. (**E**) Expression of *ALDH1A2*, *ALDH1A3*, *NANOG* and *CD133* was significantly changed in NCCIT CisR cells as determined in expression analysis by qPCR. (**F**) Analysis of stemness-related proteins in NCCIT CisR cells revealed no significant changes in Nanog, Oct-3/4, Sox17 and Sox2 levels compared to parental cells. Array spots were visualized in accordance with the manufacturer’s instructions and representative pictures are shown. 1—Sox17, 2—Oct-3/4, 3—Nanog, 4—Sox2. (**G**) Overall ALDH activity was increased in NCCIT CisR cells, as confirmed by the Aldefluor assay. The gate for ALDH+ cells was determined in relation to the DEAB control and showed the brightly fluorescent ALDH population versus the side scatter. This population was absent/decreased in the presence of DEAB. The number shown in each panel determined the percentage of ALDH+ cells. HT-29/EGFP/FUR were used for the assay setup as a positive control. * *p* < 0.05, ** *p* < 0.01, *** *p* < 0.001, **** *p* < 0.0001.

Chemoresistant variant NCCIT CisR was derived from the parental EC cells similarly as NTERA-2 CisR variant. Newly derived NCCIT CisR cells had tendency to detach from the surface of 96-well plates in adherent culture conditions and rather formed small spheroids. Therefore, we tested their chemoresistance by viability assay on day 3, when cells were still attached to the surface, or in the 3D non-adherent culture conditions on day 6. The IC_50_ value for cisplatin increased from 0.25 µg/mL in NCCIT to 0.70 µg/mL in NCCIT CisR, which is 2.8-fold increase in resistance (Figure 4A). NCCIT CisR cells were cross-resistant to carboplatin and exhibited only mild resistance to oxaliplatin (Figure 4B).

Similarly to NTERA-2 and NTERA-2 CisR cells, we were able to propagate NCCIT and NCCIT CisR cells in 3D culture conditions. Despite the fact that cisplatin-resistant NCCIT CisR cells formed significantly smaller spheroids (Figure 4C; mean spheroid volume: 0.071 ± 0.005 mm^3^ (NCCIT); 0.054 ± 0.002 mm^3^ (NCCIT CisR); *p* < 0.001), they retained significantly higher resistance (IC_50_ (NCCIT) = 0.76 µg/mL cisplatin; IC_50_ (NCCIT CisR) = 1.14 µg/mL cisplatin). Parental NCCIT spheroids were also less sensitive to cisplatin compared to both NTERA-2 (IC_50_: 0.07 µg/mL) and NTERA-2 CisR (IC_50_: 0.46 µg/mL) cell lines, what we propose to be the reason for only 1.5-fold increase in NCCIT CisR resistance (compared to 6.6-fold increase in NTERA-2 CisR spheroids). Hematoxylin and eosin staining did not show any differences in cell distribution in NCCIT and NCCIT CisR spheroids (Figure 4D).

We detected upregulation of *ALDH1A3*, *NANOG* and *CD133*; and downregulation of *ALDH1A2* genes (Figure 4E). Representative agarose gel electrophoresis of qPCR amplicons including positive controls is shown in Figure S2. Analysis of stemness-related proteins showed no significant changes in Nanog, Oct-3/4, Sox17 and Sox2 levels (Figure 4F). Overall ALDH activity was significantly increased in NCCIT CisR cells compared to parental cells (Figure 4G), recapitulating findings in NTERA-2 CisR cells.

**Figure 5:**
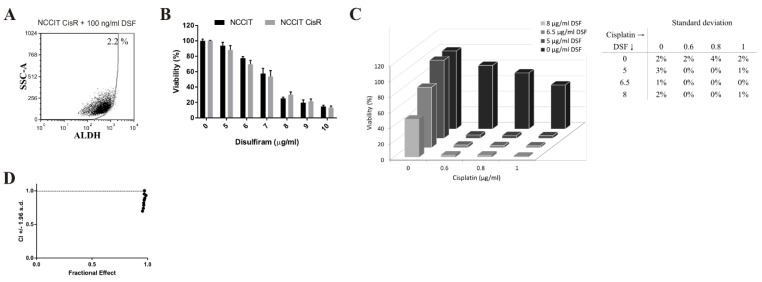
Disulfiram overcomes synergistically cisplatin resistance in NCCIT CisR 3D spheroids. (**A**) Disulfiram treatment decreased activity of ALDH in NCCIT CisR cell line compared to untreated cells in the Aldefluor assay. (**B**) Relative viability of NCCIT CisR 3D spheroids showed no significant changes compared to parental cells. Disulfiram exhibited its cytotoxicity in a dose-dependent manner. (**C**) Combination of cisplatin and disulfiram very efficiently eradicated NCCIT CisR spheroids. SD are indicated in the table. (**D**) Data obtained by luminometric assay were subsequently analyzed by Calcusyn software, and Fa-CI plots were created—CI (combination index) on the y-axis is a function of effect level (fraction affected, Fa) on the x-axis (Fa = 1—% of viable cells/100). Plots display synergism (CI < 1), additivity (CI = 1) or antagonism (CI > 1) for the entire spectrum of effects [29]. Relative viability was determined by luminescent viability assay on day 6. Values were expressed as the averages of quadruplicates ± SD.

To verify synergistic effect of the combinatorial treatment with disulfiram and cisplatin in NTERA-2 CisR cells, we used NCCIT CisR cell line as independent model to strengthen this hypothesis. Overall ALDH activity was decreased by more than 37% percent in NCCIT CisR cells after 24 h of disulfiram treatment (Figure 4G and Figure 5A). Disulfiram showed dose-depend cytotoxicity and its effect was equal on NCCIT and NCCIT CisR spheroids (Figure 5B). The combination of cisplatin and disulfiram exhibited strong synergy and even nontoxic concentration of disulfiram (5 µg/mL) very efficiently eradicated NCCIT CisR spheroids (Figure 5C). The CI indicated the strong synergistic effect of this treatment (Figure 5D).

In an independent experiment, we used another ALDH inhibitor—4-(diethylamino)-benzaldehyde (DEAB)—in combination with cisplatin to support our hypothesis and to further investigate the effect of ALDH inhibition on restoring the cisplatin sensitivity. The combination of DEAB with cisplatin decreased viability of NTERA-2 CisR and NCCIT CisR spheroids (Figure S3A,C). Fa-CI plot confirmed synergistic effect of this combination in both refractory cell lines (Figure S3B,D).

**Figure 6:**
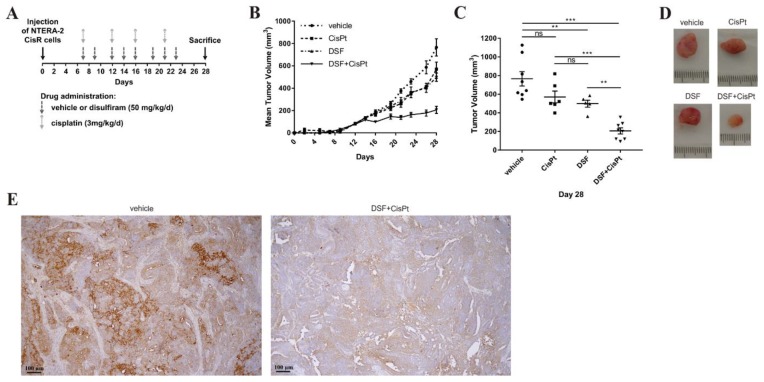
The effect of the treatment with cisplatin and disulfiram and their combination in vivo. (**A**) Outline scheme of the treatment. Cisplatin, disulfiram and vehicle were intraperitoneally administered to mice. The timing of drug administration is indicated by arrows. (**B**) Combination of cisplatin with disulfiram significantly inhibited tumor growth in vivo, *p* < 0.0001. (**C**) The tumor sizes were significantly smaller in the group of mice treated with combinatorial treatment compared to mice treated with cisplatin or disulfiram alone. (**D**) Image of representative tumors at the end of the experiment showing improved effect of combinatorial treatment. (**E**) Immunohistochemical detection of ALDH1A3 expression in xenograft germ cell tumor showed weak, moderate to strong positivity of ALDH1A3 (brown color) in teratocarcinoma of xenograft in vehicle group and weak positivity after combinatorial treatment with disulfiram and cisplatin. Original magnification 100x. ** *p* < 0.01, *** *p* < 0.001.

In order to test the effect of the combinatorial treatment on tumor growth, NTERA-2 CisR cells were injected s.c. to produce xenografts. Animals were divided into four groups according to the treatment: untreated control (eight tumors per group), group treated with cisplatin (*n* = 6), disulfiram (*n* = 5), and with combination of cisplatin and disulfiram (*n* = 8). All mice developed palpable tumors between the 5th and 7th day after the inoculation. Treatment started on day 7 according to the treatment schedule (Figure 6A). Multivariate analysis of repeated measures showed significant effect of the combinatorial treatment with disulfiram and cisplatin (*p* < 0.0001) (Figure 6B). Cisplatin alone did not significantly inhibit tumor growth of NTERA-2 CisR in vivo (the mean of tumor volume was 570 mm^3^) in comparison to control group (765 mm^3^). However, significant inhibition of the tumor growth was achieved in the animals treated with the disulfiram alone (499 mm^3^). The combination of disulfiram with cisplatin potentiated this inhibition and the mean of tumor volume in this group was almost 4-times lower (206 mm^3^) compared to control group (Figure 6C,D). According to our results showing significantly increased expression of ALDH1A3 in both resistant cell lines, we decided to further analyze expression of this isoform in tumor samples. The immunohistochemical detection of the ALDH1A3 expression in the xenograft (vehicle group) confirmed weak, moderate to strong positivity of ALDH1A3. The xenograft from the mouse treated with the combination of disulfiram and cisplatin showed weaker positivity of ALDH1A3 compared to the vehicle group (Figure 6E). These results suggest that the inhibition of ALDH activity in combination with cisplatin exerted strong antitumorigenic effect on chemoresistant TGCTs.

**Figure 7:**
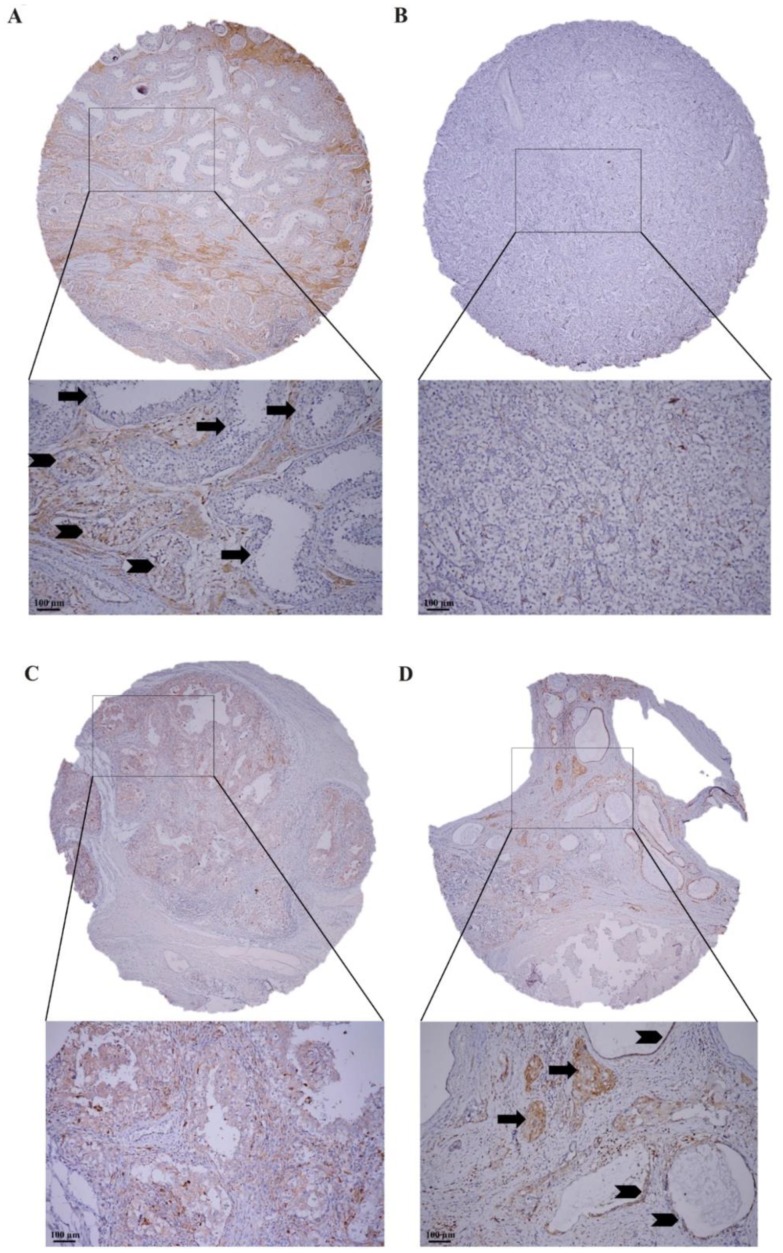
Immunohistochemical detection of the ALDH1A3 expression in testicular tissue. (**A**) Weak positivity of the ALDH1A3 (brown color) in GCNIS (arrowhead), seminiferous tubules with spermiogenesis (arrow) are ALDH1A3 negative (blue color), Leydig cells in interstitial tissue between seminiferous tubules are ALDH1A3 positive. (**B**) Negativity of the ALDH1A3 (blue color) in seminoma. (**C**) Weak positivity of the ALDH1A3 (brown color) in EC. (**D**) Moderate positivity of the ALDH1A3 (brown color) in choriocarcinoma (arrow) and epithelial structures of teratoma (arrowhead), mesenchymal structures are negative (blue color). Original magnification 40×/200×.

In order to verify the clinical significance of our findings, the expression of the ALDH1A3 was examined in tumor tissues from 216 patients before administration of systemic chemotherapy. Patients’ characteristics are summarized in the Supplementary Table S1. Majority of patients had non-seminomatous primary testicular tumor and had a good prognosis according to the International Germ Cell Cancer Collaborative Group (IGCCCG). All patients were treated with cisplatin-based chemotherapy. The analyzed cohort of the tumor tissues consisted of 52 pure seminomas (SE), 76 embryonal carcinomas (EC), 22 yolk sac tumors (YST); three choriocarcinomas (CHC) and 15 teratomas (TER) and eight germ cell neoplasia in situ (GCNIS). Forty tumor specimen were characterized as mixed germ cell tumors (Supplementary Table S2). Normal testicular tissue adjacent to the testicular tumors was available in 45 cases. The highest frequency of the ALDH1A3 expression was found in teratomas (77.8%), with decreasing trend in GCNIS (74.6%), embryonal carcinomas (71.0%), in choriocarcinomas (63.6%), yolk sac tumors (46.7%) and, at least, in seminomas (42.0%) (Figure 7A–D).

According to the weighted histoscore (HS), the ALDH1A3 expression was significantly higher in the TGCTs compared to normal testicular tissue (mean HS ± standard error of the mean [SEM] = 40.9 ± 2.9 vs. 11.3 ± 4.7, *p* < 0.0001). The highest ALDH1A3 positivity was detected in GCNIS (mean HS ± SEM = 74.6 ± 5.1). When we analyzed dichotomized ALDH1A3 expression data (absent vs. present), all of the histological subtypes of germ cell tumors exhibited significantly higher expression of the ALDH1A3 compared to normal testicular tissue (Table 1).

The association between ALDH1A3 expression in tumor cells and clinicopathological characteristics is shown in Supplementary Table S3. Metastatic sites generally did not correlate with level of ALDH1A3 expression. Moreover, there was no association found between ALDH1A3 expression and tumor primary, IGCCCG risk group, number of metastatic sites as well as S-stage. Analysis of dichotomized data revealed significant correlation only between histology of TGCTs and ALDH1A3 expression in these tumors. 

The median follow-up time was 75.5 months (0.3–186.6 months) for all 216 analyzed patients. To the date of last follow-up, 43 patients (19.9%) experienced disease progression and 29 patients (13.4%) had succumbed. We estimated no prognostic value of ALDH1A3 in analyzed cohort of patients. A survival analysis showed no differences among patients with vs. without expression of ALDH1A3 prior to treatment for DFS and OS; (HR 0.75, 95% CI 0.38–1.45, *p* = 0.416), and (HR 1.07, 95% CI 0.48–2.36, *p* = 0.875), respectively.

According to EC cell lines NTERA-2 and NCCIT used in our experiments, we performed also the exploratory sub-group analysis revealing a potential EC histology-related prognostic value of ALDH1A3. However, no statistically significant differences for DFS and OS in this sub-group of patients were found. 

This study has some limitations, including the retrospective nature of the analysis and underrepresentation of extragonadal germ cell tumors. Moreover, the semi-quantitative immunohistochemical assessment of ALDH1A3 expression and manual interpretation of IHC data might potentially contribute to the limiting factors affecting the non-significant differences for DFS and OS in these patients. In addition, ALDH1A3 expression was estimated in chemotherapy-naïve patients. Therefore, we cannot exclude the prognostic role of ALDH1A3 in chemotherapy pre-treated patients as well as in cisplatin-refractory patients.

Based on our results, we suggest that the combination of cisplatin with disulfiram very efficiently circumvents the cisplatin resistance in the chemorefractory TGCT cells. In conclusion, we propose that it is possible to augment the treatment and overcome the chemoresistance by targeting the ALDH in cisplatin-resistant solid tumors.

## 3. Discussion

The TGCTs represent a group of rare cancers with unique sensitivity to chemotherapeutic agent cisplatin. Other solid tumors also respond to cisplatin-based therapy; and this chemotherapeutic regimen represents the first line therapy in ovarian cancer and non-small cell lung cancer [9]. These tumor types share similar features associated with their unique sensitivity to alkylating agents such as: active and functional machinery for DNA recombination and re-arrangements, down-regulated nucleotide excision repair, concealed DNA damaged sites from the repair machinery, wild type p53, they are proficient in mismatch repair and devoid of BRAF mutations [4].

Many different mechanisms contribute to acquired chemoresistance [30]. Our cisplatin-resistant variants of NTERA-2 and NCCIT cell lines exhibit varying levels of cross-resistance to oxaliplatin and carboplatin, showing that the acquired resistance cannot be overcome by modified platinum-based drugs [31].

Cisplatin works as an alkylating agent and by attaching of alkyl groups to the DNA bases it mediates fragmentation of the DNA by the repair enzymes. The cross-link formed between the DNA strands prevents its synthesis and transcription, and nucleotide mispairing leads to mutations [32]. Sergent et al. have exposed colon cancer cells to suprapharmacological concentrations of cisplatin and oxaliplatin, however, they did not detect alterations in the expression of DNA mismatch repair proteins when comparing parental and drug-surviving cells [33]. Several reports investigated the alterations in the DNA repair machinery as a potential mechanism of the cisplatin resistance, however, these processes were unaffected [34]. This suggests that the resistance can be achieved via alternate mechanisms avoiding DNA repair machinery alterations.

Furthermore, we observed that NTERA-2 CisR cells were hypersensitive to DNA demethylating agent decitabine, supporting recent findings in the study showing the efficiency of the DNA methylation inhibitor guadecitabine in refractory TGCTs [35]. Importantly, platinum-resistant cells exhibiting 10-fold higher resistance in this study were derived by all-trans retinoic acid (RA)-induced differentiation of human EC cells [21], so the responsiveness to these agents was similar regardless of the procedure of resistance induction. In addition, the exposure of cisplatin-resistant TCam-2 to the demethylating agent 5-azacytidine resulted in decreased cisplatin resistance [36]. We hypothesize, that the hypomethylating agents may target cisplatin-resistant cells derived by various selection procedures in vitro and in vivo. Our NTERA-2 CisR model cells derived by prolonged exposure to cisplatin recapitulate the features of the other independently derived cisplatin-resistant TGCT preclinical models, thus being suitable for further evaluation of novel treatment options for refractory TCGT.

Recently, Schaffrath et. al. examined the activity of targeted kinase inhibitors interfering with mTOR, EGFR, Her2/Neu, VEGFR and IGF-1R signaling in sensitive and resistant TGCT cell lines [22]. Even though they demonstrated antiproliferative activity in both cell types, they did not observe improved response to cisplatin treatment in their model system [30,37]. Gutenkunst et al. suggested, that a knockdown of Oct4 in EC cell lines 2102EP and NTERA-2D1 resulted in reduced NOXA transcript and decreased hypersensitivity to cisplatin [38]. We observed the decrease (although non-significant) in Oct-3/4 expression and significantly decreased levels of Nanog and Sox2 in NTERA-2 CisR variant, too. However, *NANOG* gene expression was upregulated in both NTERA-2 CisR and NCCIT CisR cell lines at borderline significance levels.

The refractoriness to cisplatin often goes in line with the cross-resistance to other chemotherapeutic drugs which render these tumor cells refractory to many different compounds and hard-to-treat in patients. The subpopulation of chemoresistant cells often overlaps with the cells expressing cancer stem cell markers with a capability of self-renewal and propagation, therefore it is worth investigating the potential strategies targeting these markers in order to overcome the resistance [10,20,39]. 

Disulfiram, an alcohol-abuse drug, was found to have anti-cancer activity and several mechanisms of its action were described [40]. Disulfiram triggered oxidative stress by the generation of reactive oxygene species and led to apoptosis in different cancer types [41,42,43]. This drug caused cell death by apoptosis via inhibition of the proteasome activity [44,45] and autophagic cell death was shown to be also one of its anti-cancer mechanisms [46,47]. Disulfiram potently inhibited angiogenesis both in vitro and in vivo [48,49]. Skrott et al. recently reported that disulfiram is metabolized into the copper-chelating metabolite (CuET). CuET interfered with the cellular protein degradation machinery and showed the tumor suppressive effect via targeting the nuclear protein localization protein 4 (NPL4), a subunit of the p97/VCP segregase [50,51]. We analyzed the expression of *NPL4* and detected 1.12 fold downregulation of *NPL4* in NTERA-2 CisR cells compared to NTERA-2 cell line (*p* = 0.012) and no significant differences in *NPL4* expression in NCCIT and NCCIT CisR cells.

Nevertheless, disulfiram also acts by inhibiting aldehyde dehydrogenases, the family of enzymes recently associated with stem cell-like properties, including resistance to conventional chemotherapy and radiation therapy. The population of ALDH-positive CD44-positive cells was previously linked with cisplatin resistance in malignant pleural mesothelioma cells [13]. Upregulation of the ALDH1A3 isoform was correlated with acquired chemoresistance in colorectal cancer and cholangiocarcinoma [26,52]. Our newly derived cisplatin-resistant EC cell lines NTERA-2 CisR and NCCIT CisR exhibited significantly increased expression of ALDH1A3 and also higher overall ALDH activity. According to these results we suggest that ALDH, especially ALDH1A3 isoform, might be associated with cisplatin resistance in TGCTs. Multiple studies suggested repurposing disulfiram as an anti-cancer agent due to its ability to inhibit ALDH activity. Disulfiram/copper inhibited ALDH in the neurosphere population [53] and ALDH-positive non-small cell lung cancer stem cells in vitro and in vivo [54]. Moreover, disulfiram treatment led to selective decrease in the ALDH-positive cell population in triple negative breast cancer [55] and decreased ALDH1 activity in ER-positive breast cancer cells [56]. Another in vitro and in vivo data confirmed the activity of disulfiram in reversing cisplatin resistance in various experimental models [57,58,59,60,61,62]. The addition of disulfiram to a combination regimen of cisplatin and vinorelbine was well tolerated and prolonged survival in patients with newly diagnosed non-small cell lung cancer [63]. However, clinical experience in cancer patients still remains limited. In our experiments its combination with cisplatin very efficiently eradicated resistant NTERA-2 CisR and NCCIT CisR cells in the 3D culture conditions and significantly inhibited tumor growth in vivo. Our data revealed novel therapeutic combination capable of eradicating refractory TGCT.

## 4. Methods

### 4.1. Cells

Human EC cell lines NTERA-2 (ATCC^®^ CRL-1973™) and NCCIT (ATCC^®^ CRL-2073™) were purchased and used for the study within three years after purchase. NTERA-2 cells were maintained in high-glucose (4.5 g/L) Dulbecco’s modified Eagle medium (DMEM, PAA Laboratories GmbH, Pasching, Austria) containing 10% FBS (GIBCO^®^ Invitrogen, Carlsbad, CA, USA), 10.000 IU/mL penicillin (Biotica, Part. Lupca, Slovakia), 5 μg/mL streptomycin, 2.5 μg/mL amphotericin and 2 mM glutamine (PAA Laboratories GmbH). NCCIT cells were maintained in RPMI (GIBCO^®^ Invitrogen) supplemented as described above. Cells were cultivated at 37 °C in humidified atmosphere and 5% CO_2_.

Cisplatin-resistant variants of parental cell lines designated NTERA-2 CisR and NCCIT CisR were derived by propagating the cells in increasing concentrations of cisplatin (Hospira UK Ltd., Warwickshire, UK) for 6 months. Briefly, exponentially growing cells were exposed to 0.05 µg/mL cisplatin initially. When the cells started to expand, the concentrations were gradually increased to 0.1 µg/mL. Resistant variants were continuously maintained in 0.1 µg/mL cisplatin. 

Human colon cancer cell line HT-29/EGFP and its chemoresistant derivative HT-29/EGFP/FUR (kindly provided by Dr. Durinikova, Cancer Research Institute BMC SAS, Bratislava, Slovakia) were maintained in high glucose (4.5 g/L) DMEM (PAN Biotech, Aidenbach, Germany) supplemented with 10% fetal calf serum (FCS; Biochrom AG, city, Germany), 2 mM glutamine or GlutaMAX (Gibco by Life Technologies, Gaithesburg, MD, USA) 10 μg/mL gentamicin (Sandoz, Holzkirchen, Germany) and 2.5 μg/mL amphotericin [26,64]. Human mesenchymal stromal cells (MSC, kindly provided by Dr. Miklikova, Cancer Research Institute BMC SAS) used in this study were propagated in low glucose (1.0 g/L) DMEM supplemented as described above [65,66,67].

3D multicellular spheroids were prepared in quadruplicates of NTERA-2 and NTERA-2 CisR (5 × 10^3^ cells/well) or NCCIT and NCCIT CisR cells (3 × 10^3^ cells/well) and seeded into 96-well ultra-low attachment plates (Corning 7007, Corning Inc., Corning, NY, USA) in 100 μL of culture medium (as described above). Spheroids were used in the viability assays to examine the cisplatin sensitivity or in the spheroid migration assay, as described below. Representative pictures of spheroids were taken and their diameter (d) was measured using Zen 2.6 software (Carl Zeiss Microscopy GmbH, Jena, Germany). Spheroid volume was then calculated according to the formula for the volume of a sphere: volume = 1/6 × π × d.

### 4.2. Viability Assays

Chemicals were purchased from Sigma-Aldrich (Saint-Louis, MO, USA) if not stated otherwise. Quadruplicates of cells were plated at 2 × 10^3^ cells/100 μL media per well and were seeded in 96-well white-walled plates (Corning Costar Life Sciences, Amsterdam, The Netherlands) overnight. Following drugs were used: cisplatin, oxaliplatin (Fresenius Kabi Oncology Plc., Hampshire, UK), carboplatin (Fresenius Kabi Oncology Plc.) and disulfiram. For the evaluation of chemosensitivity, cells were seeded in 96-well plates overnight and treated with cisplatin (0.01–5 μg/mL), oxaliplatin (0.04–5 μg/mL), carboplatin (0.04–25 μg/mL), disulfiram (0.02–10 μg/mL) and DEAB (5–70 μg/mL). Relative viability of the cells was determined by the CellTiter-Glo™ Luminescent Cell Viability Assay (Promega Corporation, Madison, WI, USA) and evaluated by the LumiStar GALAXY reader (BMG Labtechnologies, Offenburg, Germany) after 6–7 days (NTERA-2 and NTERA-2 CisR cells) or after 3 days of treatment (NCCIT and NCCIT CisR cells). Experiments were performed in quadruplicates at least three times with similar results and the representative result is shown. Values were expressed as means ± SD and IC_50_ values were calculated by CalcuSyn 1.1 software (Biosoft, Cambridge, UK). 

Relative viability of the 3D multicellular spheroids and the efficacy of combined treatment were evaluated by the CellTiter-Glo™ 3D Cell Viability Assay (Promega Corporation). Spheroids in 96-well ultra-low attachment plates were supplemented and treated for 6 days. Medium containing drugs (cisplatin or disulfiram) was added after spheroid formation. In case of combinatorial treatment, medium containing both drugs (disulfiram + cisplatin or DEAB + cisplatin) was added at the same time after spheroid formation as the simultaneous treatment. Cells were cultured for next 6 days and combinational effect of drugs was calculated according to Chou [29]. Briefly, CI was computed for every affected fraction (Fa, proportion of dead cells): CI < 1 represents synergism, CI = 1 represents additivity, antagonism is defined with CI > 1. CalcuSyn software was used for analysis [68].

### 4.3. 3D Spheroid Migration Assay

3D multicellular spheroids were prepared in octaplicates of 5 × 10^3^ NTERA-2 or NTERA-2 CisR cells as described above. After 72 h of cultivation, spheroids were placed on top of a conventional cell culture 96-well plate (CytoOne, USA Scientific, Inc., Ocala, FL, USA). After attachment of the spheroid to the plastic surface, the cells started to migrate and the area of attachment was scanned by IncuCyte ZOOM™ Kinetic Imaging System for next 24 h.

### 4.4. Caspase-3/7 Assay

Quadruplicates of 3 × 10^4^ cells per well were seeded in 96-well white-walled plates overnight. Cisplatin (8 μg/mL) diluted in culture media was added to the cells for 6 or 12 h and a caspase-3/7 activity was determined by the Caspase-Glo^®^ 3/7 Assay (Promega Corporation) on LUMIstar GALAXY reader at indicated timepoints. Values were determined as mean values of RLU ± SD. Same procedure of cultivation was used to determination of cell viability by the CellTiter-Glo™ Luminescent Cell Viability Assay as described above.

### 4.5. α-F-Actin Immunostaining

Ten thousand cells grown on microscopic slides for 72 h were fixed with 4% paraformaldehyde in PBS for 15 min at room temperature and permeabilized with 0.05% Triton-X100 in PBS for 15 min. After overnight incubation with anti-F-actin rhodamine-conjugated antibody (1:500; Molecular Probes, Waltham, MA, USA), nuclei were counterstained with DAPI (1:500). Staining patterns were analyzed with a Zeiss fluorescent microscope (AxioImager. Z2, Metafer, MetaSystems GmbH, Altlußheim, Germany) using Isis upgrade software for Metafer (MetaSystems GmbH, Altlußheim, Germany).

### 4.6. Hematoxylin and Eosin Staining

3D multicellular spheroids were prepared as described above and harvested from 96-well culture plates after 3 days. All spheroids were immediately fixed for 3 h in Carnoy’s solution, transferred to 96% ethanol, next to the solution of ethanol: chloroform (1:1), and then to chloroform. Spheroids were embedded in paraffin blocks, histological sections (5 μm) were prepared using a microtome and subsequently stained with hematoxylin and eosin. Digital images were captured and analyzed with Axiovert 40C Zeiss microscope using the Zen 2.6 software. 

### 4.7. Proteome Profiler 

Proteome profile analysis of stem cell markers was done by the Human Pluripotent Stem Cell Array Kit (R&D Systems™, Minneapolis, MN, USA). ImageJ software (NIH, Bethesda, MD, USA) was used for the quantitative evaluation; pixel density was determined and calculated.

Untreated cell lines were dissociated, counted and lysed at a concentration of 1 × 10^7^ cells/mL in lysis buffer at 2–8 °C for 30 min. Protease inhibitors were added to the lysis buffer at recommended concentration (Complete Protease Inhibitor Cocktail Tablets, Roche Diagnostics Deutschland GmbH, Mannheim, Germany). Protein lysate (200 μg total protein) was loaded on the membranes with blotted antibodies and evaluated as recommended by the manufacturer.

### 4.8. Gene Expression

Cultured cells were collected by trypsinization and total RNA was isolated by NucleoSpin^®^ RNA II (Macherey-Nagel, Düren, Germany) and treated with RNase-free DNase (Qiagen, Hilden, Germany). Total RNA was subjected to control PCR to confirm the absence of genomic DNA contamination. RNA concentration and quality were determined by gel electrophoresis and spectrophotometrically at 260/280 nm using the NanoDrop ND-1000 Spectrophotometer (Thermo Scientific, Waltham, MA, USA).

RNA was reverse transcribed with RevertAid™ H minus First Strand cDNA Synthesis Kit (Thermo Fisher Scientific Inc., Waltham, MA, USA). For qPCR was used following protocol: activation step at 95 °C for 2 min, 40 cycles of denaturation at 95 °C for 15 s, 30 s annealing and polymerization at 60 °C and plate read for 5 s at 71 °C, followed by melt cycle. The PCR reaction mixture (15 μL) contained 1 μL cDNA (100 ng), 0.4 μL of the respective specific primers (10 pmol/μL), 6.1 μL water and 7.5 μL GoTaq^®^ qPCR Master Mix (Promega Corporation). qPCR reaction was performed on CFX96™ Real-Time PCR Detection System (BIO-RAD Laboratories, Hercules, CA, USA) and analyzed by Bio-Rad CFX Manager software version 1.6. *HPRT1* or *ACTB* gene expressions were taken as endogenous reference. Relative gene expression was calculated using the 2^–ΔΔCt^ method. The results were reported as the n-fold change in gene of interest expression in the resistant cell line normalized to the endogenous control (*HPRT1* or *ACTB*) and relative to the control group (= 1). Data represent mean ± SEM of three independent experiments. The significance of fold changes in gene expression between groups was analyzed using Student’s t-test applied to the ΔCt values. Horizontal electrophoresis using 4% MetaPhor Agarose (Lonza, Rockland, ME, USA) and GeneRuler 50 bp DNA Ladder (Thermo Fisher Scientific Inc.) was used for qualitative assessment of amplicons. The primer sequences used for expression analysis are listed in Table 2.

### 4.9. Aldefluor Assay

The ALDEFLUOR^TM^ Kit (Stem Cell Technologies, Vancouver, BC, Canada) was used to detect intracellular enzyme activity of ALDH. Samples were prepared according to manufacturer’s instructions and ALDH activity was analyzed using BD Canto II Cytometer (Becton Dickinson, Franklin Lakes, NJ, USA). Dead cells were excluded from the analysis based on 4′,6-diamidino-2-phenyl-indole (DAPI) staining. Data were analyzed by the FCS Express program (De Novo Software, Glendale, CA, USA).

### 4.10. Animal Studies

Six- to 8-week-old athymic nude mice (Balb/c-nu/nu, Charles River, Sulzfeld, Germany) or SCID beige mice (CD17 Cg-Prkdscid Lystbg/Crl) were used in accordance with institutional guidelines under approved protocols. Project was approved by the Institutional Ethic Committee and by the national competence authority (State Veterinary and Food Administration of the Slovak Republic), registration No. Ro 1030/18-221 in compliance with the Directive 2010/63/EU and the Regulation 377/2012 on the protection of animals used for scientific purposes. It was performed in the approved animal facility (license No. SK UCH 02017).

For the tumorigenicity test, suspension of 2 × 10^5^ NTERA-2 and NTERA-2 CisR cells in 100 µL of extracellular matrix (ECM) mixture 1:1 (50 µL serum free DMEM medium, 50 µL ECM) was injected s.c. into the flank of SCID beige mouse, in total five tumors per group. 

In an independent study of disulfiram in vivo, nude mice were used. Suspension of 2 × 10^5^ NTERA-2 and NTERA-2 CisR in 100 µL of ECM mixture 1:1 (50 µL serum free DMEM medium, 50 µL ECM) was injected s.c. into the flanks, in total two injections per animal. Mice were divided into four groups according to the treatment: cisplatin i.p./disulfiram i.p./disulfiram and cisplatin i.p./untreated controls. 

In both experiments, tumors were measured by caliper and volume was calculated according to the formula for the volume of ellipsoid: volume = 0.52 × ((width + length)/2)^3^. Animals were sacrificed at the point when the tumors exceeded 1 cm in diameter. The results were evaluated as the mean of tumor volume or tumor weight.

### 4.11. Patient Data

Two hundred and sixteen (216) patients diagnosed with the TGCTs and treated from October 1995 to September 2013 in the National Cancer Institute of Slovakia, Bratislava, Slovakia and St. Elisabeth Cancer Institute, Bratislava, Slovakia, were included into the study (Protocol IZLO1, Chair: M. Mego). Patients with a concurrent malignancy other than non-melanoma skin cancer in the previous 5 years were excluded from the study. In all patients, data regarding tumor histological subtype were recorded and compared with the ALDH1A3 expression. The Institutional Review Board of the National Cancer Institute, Bratislava, Slovakia approved this retrospective study and a waiver of patient consent was granted.

### 4.12. Tumor Pathology

Pathological review was conducted at the Department of Pathology, Faculty of Medicine, Comenius University, Bratislava, Slovakia by pathologist experienced in TGCTs pathology (Z.C.).

### 4.13. Diagnosis and Tissue Samples

Tumor tissue, samples with germ cell neoplasias in situ (GCNIS), and normal testicular tissue were evaluated in all cases, when available. The study included tumor specimens from 216 patients prior to the administration of systemic therapy. Primary testicular tumor specimens were obtained in 208 (96.3%) patients. Biopsies of abdominal and mediastinal metastatic masses were performed in 8 (3.7%) cases. 

The TGCTs were classified according to the World Health Organization criteria [69]. Since the normal testicular biopsies from non-cancer patients were not available for our analysis, we used normal tissue adjacent to testicular tumor for the ALDH1A3 expression evaluation, as described in previous studies [70,71,72]. Non-neoplastic adjacent testicular tissue was available for the ALDH1A3 expression evaluation in 45 specimens.

### 4.14. Tissue Microarray Construction

According to the tumor histology, one or two representative tumor areas from each histological subtype of germ cell tumor (from 1–6 cores from each tumor) were identified on haematoxylin- and eosin-stained sections. Samples from normal testicular tissue and germ cell neoplasia in situ were also marked, if present. Sections were matched to their corresponding wax blocks (the donor blocks), and 3-mm diameter cores of the tumor were removed from these donor blocks with the multipurpose sampling tool Harris Uni-Core (Sigma-Aldrich, Steinheim, Germany) and inserted into the recipient master block. The recipient block was cut into 5-μm sections that were transferred to coated slides.

### 4.15. Immunohistochemical Staining

Deparaffinized slides were rehydrated in phosphate buffered saline solution (10 mM, ph 7.2). The tissue epitopes were demasked using the automated water bath heating process in Dako PT Link (Dako, Glostrup, Denmark); the slides were incubated in TRIS-EDTA retrieval solution (10 mM TRIS, 1 mM EDTA pH 9.0) at 98 °C for 20 min. The slides were subsequently incubated for 1 h at room temperature with the primary rabbit polyclonal antibody against ALDH1A3 (Abcam, AB175844) diluted 1:100 in Dako REAL antibody diluent (Dako) and immunostained using anti-mouse/anti-rabbit immuno-peroxidase polymer (EnVision FLEX/HRP, Dako) for 30 minutes at room temperature, according to the manufacturer’s instructions. For visualization, the slides were reacted with diaminobenzidine substrate-chromogen solution (DAB, Dako) for 5 minutes. Finally, the slides were counterstained with haematoxylin.

### 4.16. Immunohistochemical Scoring

ALDH1A3 expression was scored using weighted histoscore (HS) according to the extent of cell staining as well as staining intensity [73]. Briefly, the portion of positive cells was estimated on a scale of 0–100%. The average intensity of the positively stained cells was given a score of 0 to 3 (0 = no staining; 1 = weak; 2 = intermediate; and 3 = strong staining). Weighted histoscore was then calculated by multiplying the percentage score by the intensity score, to yield a minimum value of 0 and a maximum value of 300. ALDH1A3 expression was also stratified as negative or positive (any staining).

### 4.17. Statistical Analysis

For the statistical analysis of data from in vitro experiments, the normality assumption hypothesis was tested using Shapiro-Wilk test. Differences between two groups in individual time points were assessed by Student’s t-test or Mann-Whitney U test depending on normality of the data in GraphPad Prism^®^ software (GraphPad Inc., La Jolla, CA, USA). The migratory capacity and the effects of monotherapies and combination treatment in vivo were tested using multivariate analysis. The General linear model was applied for repeated measures with Greenhouse-Geisser correction if violation of sphericity was assumed.

The patients’ characteristics were tabulated as mean or median (range) values for continuous variables and frequency (percentage) for categorical variables, respectively. Because of the distribution of the ALDH1A3 HS was significantly different from the normal distribution (Shapiro-Wilk test), non-parametric tests were used for the statistical analysis. Mann–Whitney U test was used in analyses of differences in distributions of ALDH1A3 expression between the two groups of patients, while Fisher’s exact test or the χ^2^ test were performed when ALDH1A3 expression was categorized as ‘absent’ or ‘present’ based on the aforementioned criteria. A median follow-up period was estimated as a median observation time among all patients and among those still alive at the time of their last follow-up. DFS was calculated from the date of orchiectomy or the date of tumor biopsy to the date of the disease progression or death or the date of the last adequate follow-up. OS was calculated from the date of orchiectomy or the date of tumor biopsy to the date of death or last the follow-up. DFS and OS were estimated using Kaplan-Meier product limit method and compared between groups by log-rank test. Statistical analysis of data was performed using IBM SPSS statistics version 23.0 software for Windows (IBM). The *p*-values with *p* < 0.05 were considered to be statistically significant.

## 5. Conclusions

In summary, we describe here novel model for the refractory TGCTs which exhibit features recapitulating the other preclinical findings. We have demonstrated feasibility of the 3D spheroid cultures for evaluation of the drug efficiencies. Moreover, we have identified the ALDH being overexpressed in the cisplatin-resistant variants. Importantly, we identified that the ALDH inhibition by disulfiram augmented cisplatin toxicity in vitro and in vivo. This study showed for the first time significant differences in the ALDH1A3 expression in different histological subtypes of testicular tumors compared to normal testicular tissue. We suggest here that disulfiram, as an ALDH inhibitor, is suitable for the combination therapy in order to achieve antitumor effect in the refractory TGCT in patients.

## Figures and Tables

**Table 1:** Aldh1a3 Expression in Different Histological Subtypes of Germ Cell Tumors.

		Expression of ALDH1A3								
Histologic Subtype	N	Mean Score	SEM	Median	*p*-Value ^c^	Absent		Present		*p*-Value ^c^
						N	%	N	%	
Normal tissue adjacent to testicular tumors	45	11.3	4.7	0.0	NA	39	86.7	6	13.3	NA
Testicular germ cell tumors	216	40.9	2.9	20.0	<0.0001	64	29.6	152	70.4	<0.0001
GCNIS	59	74.6	5.1	100.0	<0.0001	15	25.4	44	74.6	<0.0001
Seminoma	69	10.1	3.0	0.0	0.008	40	58.0	29	42.0	0.002
Embryonal carcinoma	107	28.5	3.2	15.0	<0.0001	31	29.0	76	71.0	<0.0001
Yolk sac tumor	30	9.8	5.0	0.0	0.007	16	53.3	14	46.7	0.003
Choriocarcinoma	11	39.5	10.2	20.0	0.001	4	36.4	7	63.6	0.002
Teratoma	36	49.6	6.9	20.0	<0.0001	8	22.2	28	77.8	<0.0001

^c^ compared to normal tissue adjacent to testicular tumors.

**Table 2:** Sequences of Primers Used for Expression Analysis.

Gene	Forward Primer (5′ to 3′)	Reverse Primer (5′ to 3′)	Product Size
ALDH1A1	TTGGAATTTCCCGTTGGTTA	CTGTAGGCCCATAACCAGGA	182 bp
ALDH1A2	AGGGCAGTTCTTGCAACCATGGAA	CACACACTCCAATGGGTTCATGTC	193 bp
ALDH1A3	GCCCTTTATCTCGGCTCTCT	CGGTGAAGGCGATCTTGT	133 bp
ALDH1B1	GCCCCTGTTCAAGTTCAAG	CCTTAAACCCTCCAAATGG	194 bp
OCT4	ACATCAAAGCTCTGCAGAAAGAACT	CTGAATACCTTCCCAAATAGAACCC	133 bp
NANOG	CAAAGGCAAACAACCCACTT	ATTGTTCCAGGTCTGGTTGC	346 bp
SOX2	GGAAAGTTGGGATCGAACAA	GCGAACCATCTCTGTGGTCT	145 bp
ABCG2	CGGGTGACTCATCCCAACAT	CAGGATCTCAGGATGCGTGC	75 bp
CD133	TGGATGCAGAACTTGACAACGT	ATACCTGCTACGACAGTCGTGGT	133 bp
endosialin	CGCAGTTGCGAGGACCCCTG	ATCTGCTGGCACACACCGGC	170 bp
MRP1	GCGAGTGTCTCCCTCAAACG	TCCTCACGGTGATGCTGTTC	118 bp
HPRT1	GGACTAATTATGGACAGGACT	GCTCTTCAGTCTGATAAAATCTAC	195 bp
ACTB	GGACTTCGAGCAAGAGATGG	AGCACTGTGTTGGCGTACAG	235 bp

## Supplementary Materials

The following are available online at https://www.mdpi.com/2072-6694/11/9/1224/s1, Figure S1, Cisplatin resistance correlates with increased migratory capacity in NTERA-2 CisR cells. A Time lapse of NTERA-2 and NTERA-2 CisR cell adhesion and migration out of formed spheroids acquired every 8 h. NTERA-2 CisR cells formed tight rounded spheroids with evenly distributed cells attaching to the surface. B Quantitative evaluation of spheroid area did not show significant changes in migratory capacity of NTERA-2 CisR cells compared to parental cells. Data are expressed as means ± SD. C Migration of NTERA-2 and NTERA-2 CisR cells out of the spheroids was imaged 96 h post transfer from non-adherent to adherent conditions. NTERA-2 speroids already started to fall apart, magnification 250x. Figure S2, Qualitative assessment of qPCR amplicons. MSC were used as a positive control for the *OCT4*, *ABCG2*, endosialin, *MRP1*; NCCIT for the *SOX2*, HT-29/EGFP for the *ALDH1A1*, *NANOG*, *CD133*, and *HPRT1*, chemoresistant HT-29/EGFP/FUR for the *ALDH1A2*, *ALDH1A3* and *ALDH1B1* expression. Amplicon sizes are listed in Table 2. Figure S3, DEAB resensitizes chemoresistant NTERA-2 CisR and NCCIT CisR spheroids to cisplatin. A, C DEAB in combination with cisplatin showed synergistic effect and decreased viability of NTERA-2 CisR and NCCIT CisR spheroids 6 days post treatment start. Relative viability was determined by luminescent viability assay. Values are expressed as the averages of quadruplicates ± SD. B, D Data obtained by luminometric assay were subsequently analyzed by Calcusyn software, and Fa-CI plots were created – CI on the y-axis is a function of effect level (fraction affected, Fa) on the x-axis (Fa = 1 - % of viable cells/100). Plots display synergism (CI < 1), additivity (CI = 1) or antagonism (CI > 1) for the entire spectrum of effects [29]. Table S1, Patients characteristics (*n* = 216). Table S2, Composition of mixed TGCTs (*N* = 40). Supplementary Table S3. Patients’ characteristics according to the expression of ALDH1A3 (*n* = 216)
