# Supplementary material for: Disulfiram Overcomes Cisplatin Resistance in Human Embryonal Carcinoma Cells
Source: Cancers (Basel). 2019 Aug 22;11(9):1224. doi: 10.3390/cancers11091224 (PMC6769487; doi:10.3390/cancers11091224)
Supplement: Supplementary file 1 [file cancers-11-01224-s001.zip › Supplementary Table S1.docx]

**Supplementary Table S1.** Patients characteristics (n=216)

|  | **N = 216** | **%** |
| --- | --- | --- |
| **Age (years)** |  |  |
| Median (range) | 31 (16-67) | NA |
| **Histology**^a^ |  |  |
| Pure seminoma | 40 | 18.5 |
| Non-seminoma or mixed GCT | 173 | 80.1 |
| **Primary tumor** |  |  |
| Gonadal | 208 | 96.3 |
| Primary retroperitoneal | 6 | 2.8 |
| Primary mediastinal | 2 | 0.9 |
| **IGCCCG risk group** |  |  |
| Good risk | 165 | 76.4 |
| Intermediate risk | 25 | 11.6 |
| Poor risk | 26 | 12.0 |
| **Sites of metastases^b- 1 pts NA^** |  |  |
| Retroperitoneum | 149 | 69.0 |
| Mediastinum | 20 | 9.3 |
| Lungs | 48 | 22.2 |
| Liver | 11 | 5.1 |
| Other | 11 | 5.1 |
| Non-pulmonary visceral metastases | 14 | 6.5 |
| **No. of metastatic sites^b- 1 pts NA^** |  |  |
| 0 | 57 | 26.4 |
| 1-2 | 130 | 60.2 |
| > 3 | 28 | 13.0 |

^a^ in three patients therapy started without histological confirmation of disease (neoadjuvant therapy)

^b^ data not available in one patient
