# Supplementary material for: Disulfiram Overcomes Cisplatin Resistance in Human Embryonal Carcinoma Cells
Source: Cancers (Basel). 2019 Aug 22;11(9):1224. doi: 10.3390/cancers11091224 (PMC6769487; doi:10.3390/cancers11091224)
Supplement: Supplementary file 1 [file cancers-11-01224-s001.zip › Supplementary Table S2.docx]

**Supplementary Table S2.** Composition of mixed TGCTs (N =40)

| **Histological subtype** | | | | | **Number  of patients** |
| --- | --- | --- | --- | --- | --- |
| EC |  |  |  | TER | 12 |
| EC | SE |  |  |  | 11 |
| EC |  | YST |  |  | 3 |
| EC |  |  | CHC | TER | 3 |
|  | SE |  | CHC |  | 2 |
|  |  | YST |  | TER | 2 |
| EC |  |  | CHC |  | 1 |
|  | SE | YST |  |  | 1 |
|  | SE |  |  | TER | 1 |
|  |  | YST | CHC |  | 1 |
|  |  |  | CHC | TER | 1 |
| EC | SE |  |  | TER | 1 |
|  | SE | YST |  | TER | 1 |

EC, embryonal carcinoma; SE, seminoma; YST, yolk sac tumor; CHC, choriocarcinoma; TER, teratoma; GCNIS, germ cell neoplasia *in situ*
