# Supplementary material for: Disulfiram Overcomes Cisplatin Resistance in Human Embryonal Carcinoma Cells
Source: Cancers (Basel). 2019 Aug 22;11(9):1224. doi: 10.3390/cancers11091224 (PMC6769487; doi:10.3390/cancers11091224)
Supplement: Supplementary file 1 [file cancers-11-01224-s001.zip › Supplementary Table S3.docx]

**Supplementary Table S3.** Patients’ characteristics according to the expression of ALDH1A3 (n=216)

|  |  | **ALDH1A3** | | | | | | | | |
| --- | --- | --- | --- | --- | --- | --- | --- | --- | --- | --- |
| **Variable** | **N** | **Mean** | **SEM** | **Median** | **p-value** | **Present** | | **Absent** | | **p-value** |
|  |  |  |  |  |  | **N** | **%** | **N** | **%** |  |
| **All patients** | 216 | 40.9 | 3.0 | 20.0 | NA | 152 | 70.4 | 64 | 29.6 | NA |
| **Histology ^a^** |  |  |  |  |  |  |  |  |  |  |
| Pure seminoma | 40 | 35.0 | 7.0 | 7.5 | 0.297 | 23 | 57.5 | 17 | 42.5 | **0.055** |
| Non-seminoma | 173 | 42.4 | 3.4 | 20.0 |  | 127 |  | 46 |  |  |
| **Tumor primary** |  |  |  |  |  |  |  |  |  |  |
| Testicular/retroperitoneal | 214 | 41.3 | 3.0 | 20.0 | 0.076 | 151 | 70.6 | 63 | 29.4 | 0.506 |
| Mediastinal | 2 | 0.0 | 31.3 | 0.0 |  | 1 | 50.0 | 1 | 50.0 |  |
| **IGCCCG risk group** |  |  |  |  |  |  |  |  |  |  |
| Good | 165 | 43.6 | 3.4 | 20.0 | 0.108 | 113 | 68.5 | 52 | 31.5 | 0.298 |
| Intermediate/Poor | 51 | 32.2 | 6.2 | 10.0 |  | 39 | 78.0 | 12 | 23.5 |  |
| **Number of metastatic sites ^b^** |  |  |  |  |  |  |  |  |  |  |
| 0 | 57 | 40.5 | 5.9 | 25.0 | 0.429 | 38 | 66.7 | 19 | 33.3 | 0.691 |
| 1 to 2 | 130 | 39.9 | 3.9 | 10.0 |  | 93 | 71.5 | 37 | 28.5 |  |
| ≥ 3 | 28 | 47.9 | 8.4 | 30.0 |  | 21 | 75.0 | 7 | 25.0 |  |
| **Retroperitoneal LN metastases^b- 1 pts NA^** |  |  |  |  |  |  |  |  |  |  |
| Absent | 66 | 38.2 | 5.6 | 22.5 | 0.434 | 43 | 65.2 | 23 | 34.8 | 0.257 |
| Present | 149 | 42.9 | 3.6 | 15.0 |  | 109 | 73.2 | 40 | 28.6 |  |
| **Mediastinal LN metastases ^b^** |  |  |  |  |  |  |  |  |  |  |
| Absent | 195 | 41.7 | 3.2 | 20.0 | 0.458 | 136 | 69.7 | 59 | 30.3 | 0.443 |
| Present | 20 | 37.5 | 9.9 | 15.0 |  | 16 | 80.0 | 4 | 20.0 |  |
| **Lung metastases ^b^** |  |  |  |  |  |  |  |  |  |  |
| Absent | 167 | 40.7 | 3.5 | 20.0 | 0.443 | 118 | 70.7 | 49 | 29.3 | 1.00 |
| Present | 48 | 43.3 | 6.4 | 17.5 |  | 34 | 70.8 | 14 | 29.2 |  |
| **Liver ^b^** |  |  |  |  |  |  |  |  |  |  |
| Absent | 204 | 40.9 | 3.1 | 20.0 | 0.456 | 146 | 71.6 | 58 | 28.4 | 0.307 |
| Present | 11 | 49.1 | 13.4 | 60.0 |  | 6 | 54.5 | 5 | 45.5 |  |
| **Brain ^b^** |  |  |  |  |  |  |  |  |  |  |
| Absent | 213 | 41.6 | 3.0 | 20.0 | 0.321 | 151 | 70.9 | 62 | 29.1 | 0.503 |
| Present | 2 | 10.0 | 31.4 | 10.0 |  | 1 | 50.0 | 1 | 50.0 |  |
| **Non-pulmonary visceral metastases^b- 1 pts NA^** |  |  |  |  |  |  |  |  |  |  |
| Absent | 201 | 41.2 | 3.1 | 20.0 | 0.457 | 143 | 71.1 | 58 | 28.9 | 0.558 |
| Present | 14 | 42.9 | 11.9 | 17.5 |  | 9 | 64.3 | 5 | 35.7 |  |
| **S – stage ^c^** |  |  |  |  |  |  |  |  |  |  |
| 0-1 | 168 | 42.6 | 3.4 | 20.0 | 0.639 | 114 | 67.9 | 54 | 32.1 | 0.103 |
| 2-3 | 46 | 37.1 | 6.5 | 10.0 |  | 37 | 80.4 | 9 | 19.6 |  |

^a^ in three patients therapy started without histological confirmation of disease (neoadjuvant therapy), ^b^ data not available in one patient, ^c^ data not available in two patients
