# Supplementary figures and images for: Disulfiram Overcomes Cisplatin Resistance in Human Embryonal Carcinoma Cells
Source: Cancers (Basel). 2019 Aug 22;11(9):1224. doi: 10.3390/cancers11091224 (PMC6769487; doi:10.3390/cancers11091224)

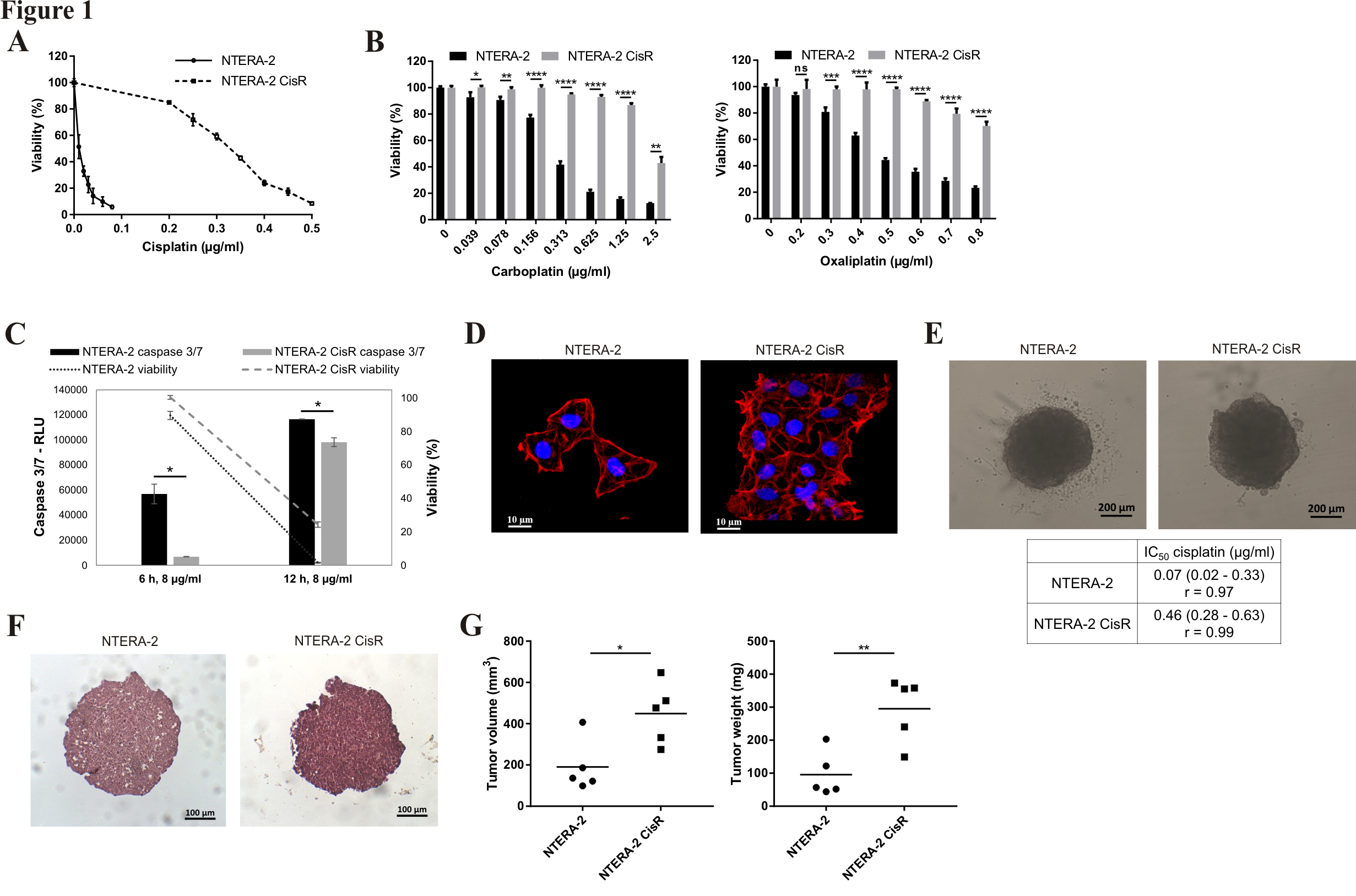

Supplement: Supplementary file 1 [file cancers-11-01224-s001.zip › fig 1.tif]

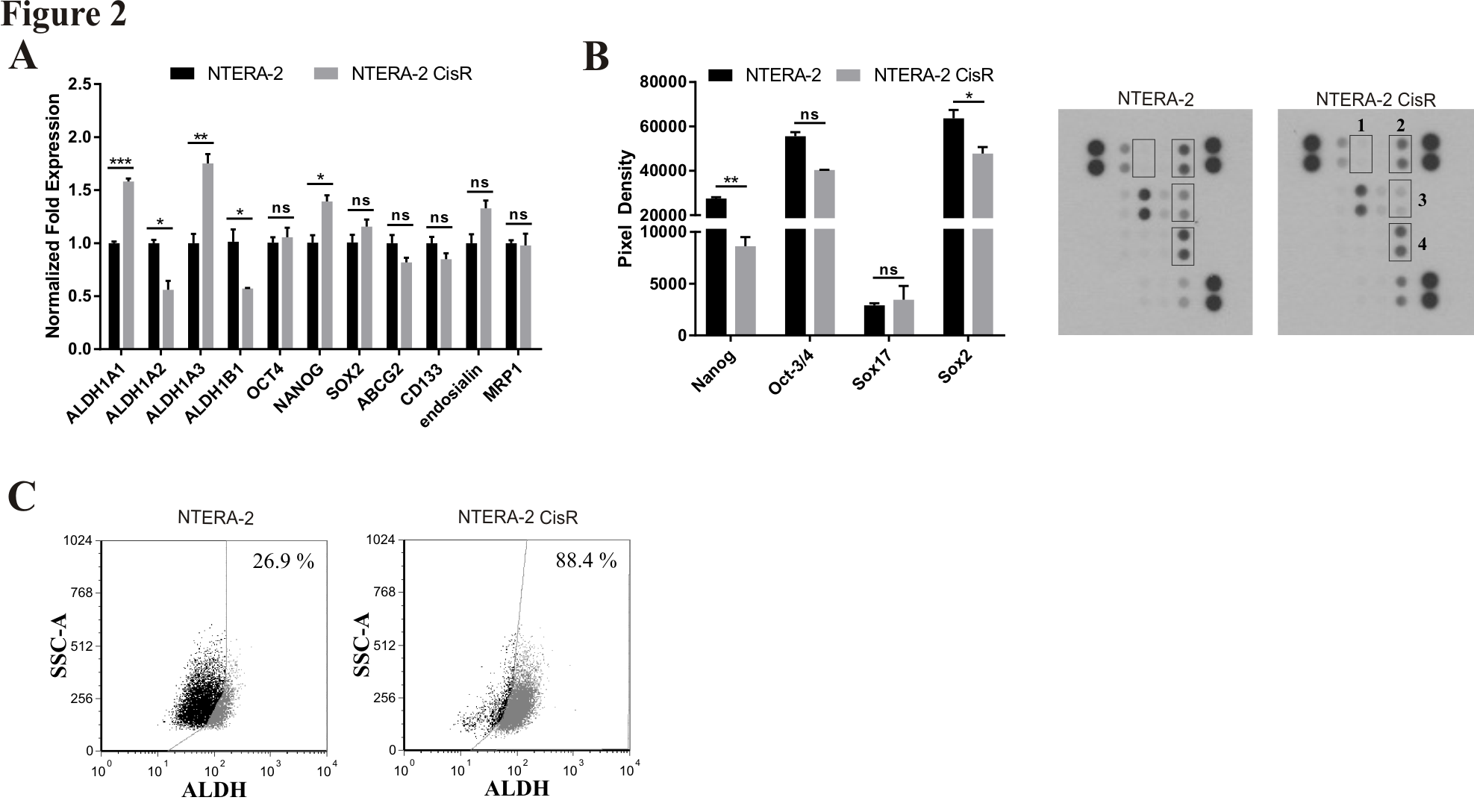

Supplement: Supplementary file 1 [file cancers-11-01224-s001.zip › fig 2.tif]

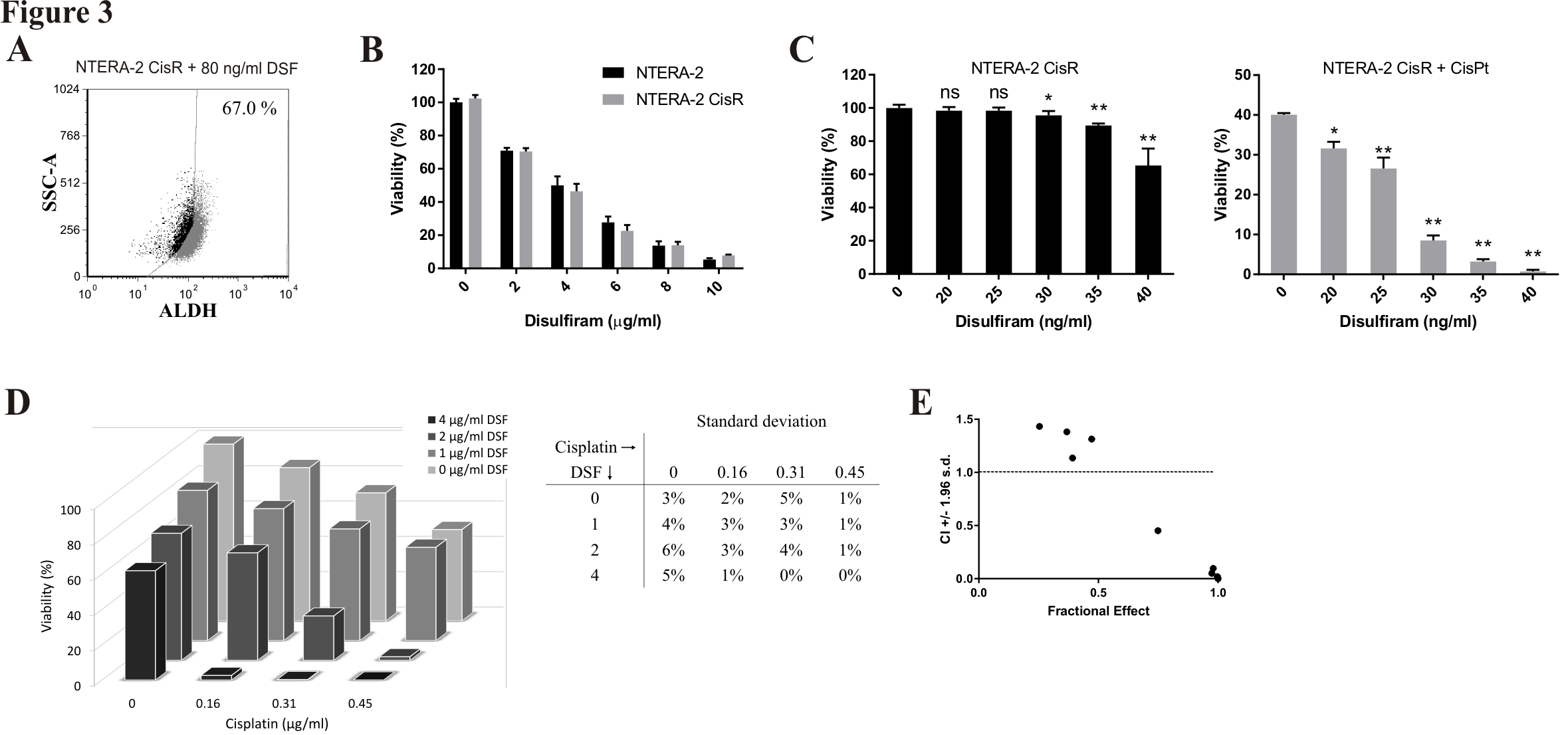

Supplement: Supplementary file 1 [file cancers-11-01224-s001.zip › fig 3.tif]

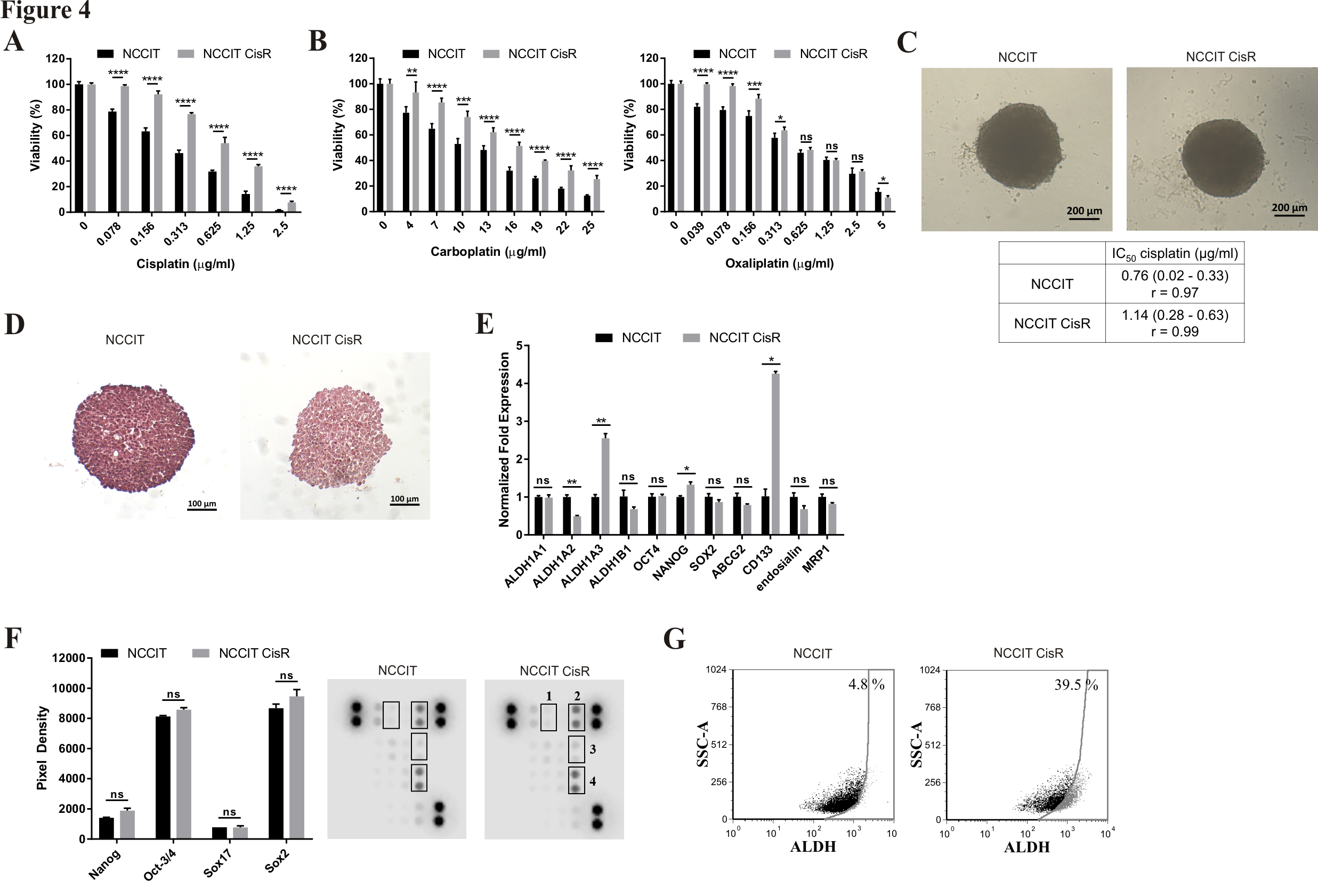

Supplement: Supplementary file 1 [file cancers-11-01224-s001.zip › fig 4.tif]

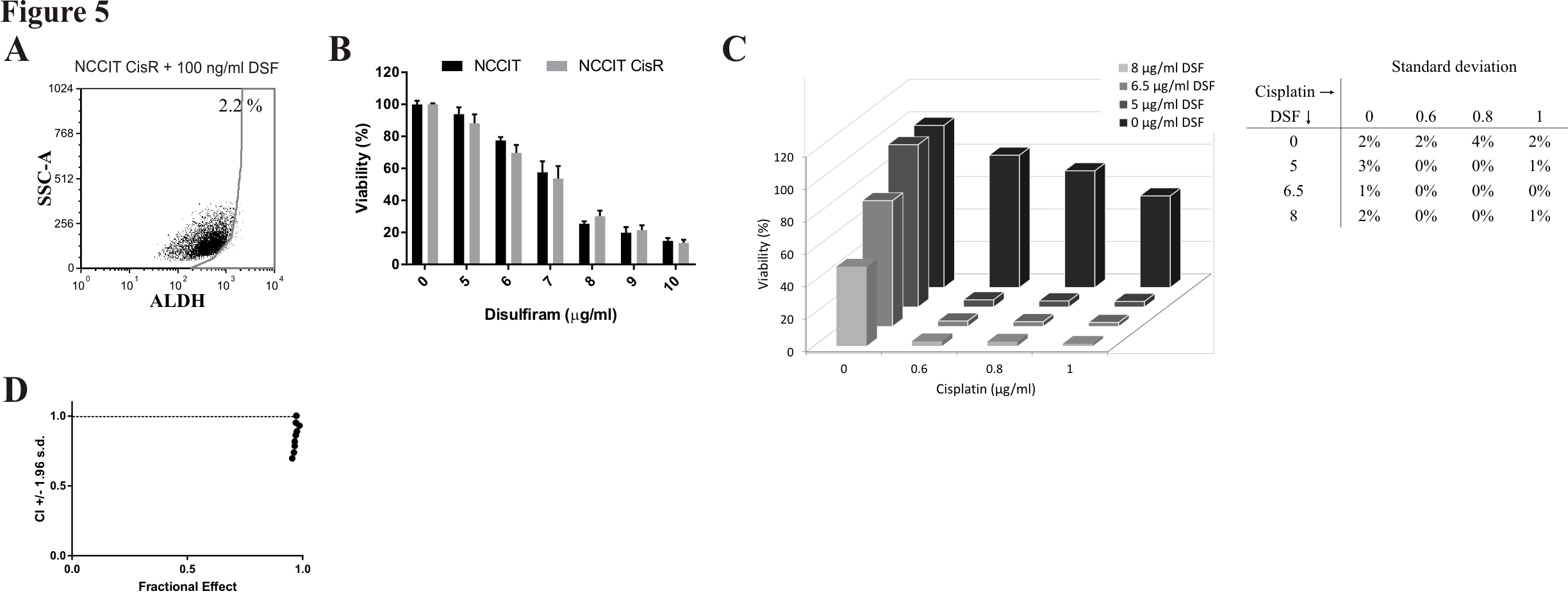

Supplement: Supplementary file 1 [file cancers-11-01224-s001.zip › fig 5.tif]

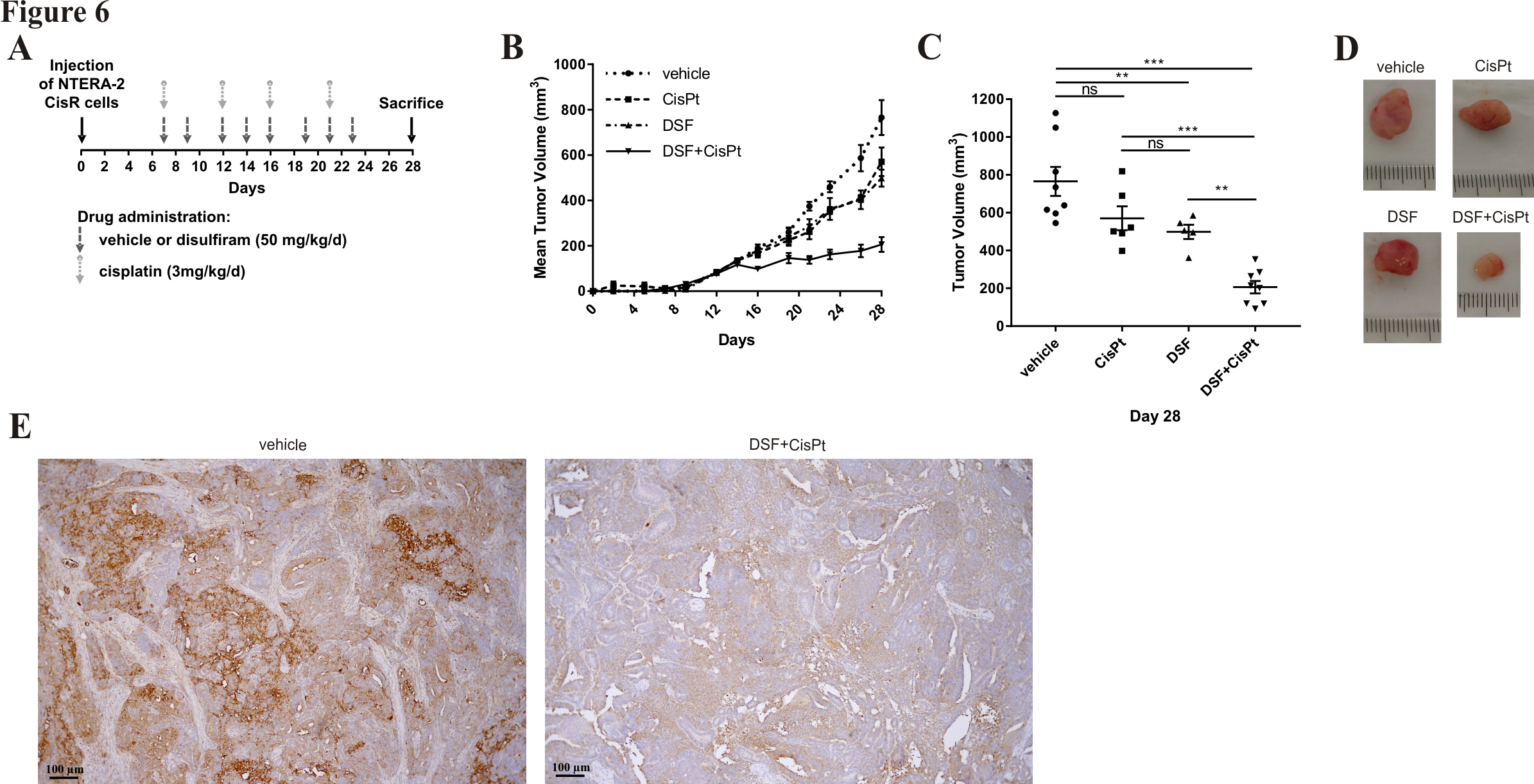

Supplement: Supplementary file 1 [file cancers-11-01224-s001.zip › fig 6.tif]

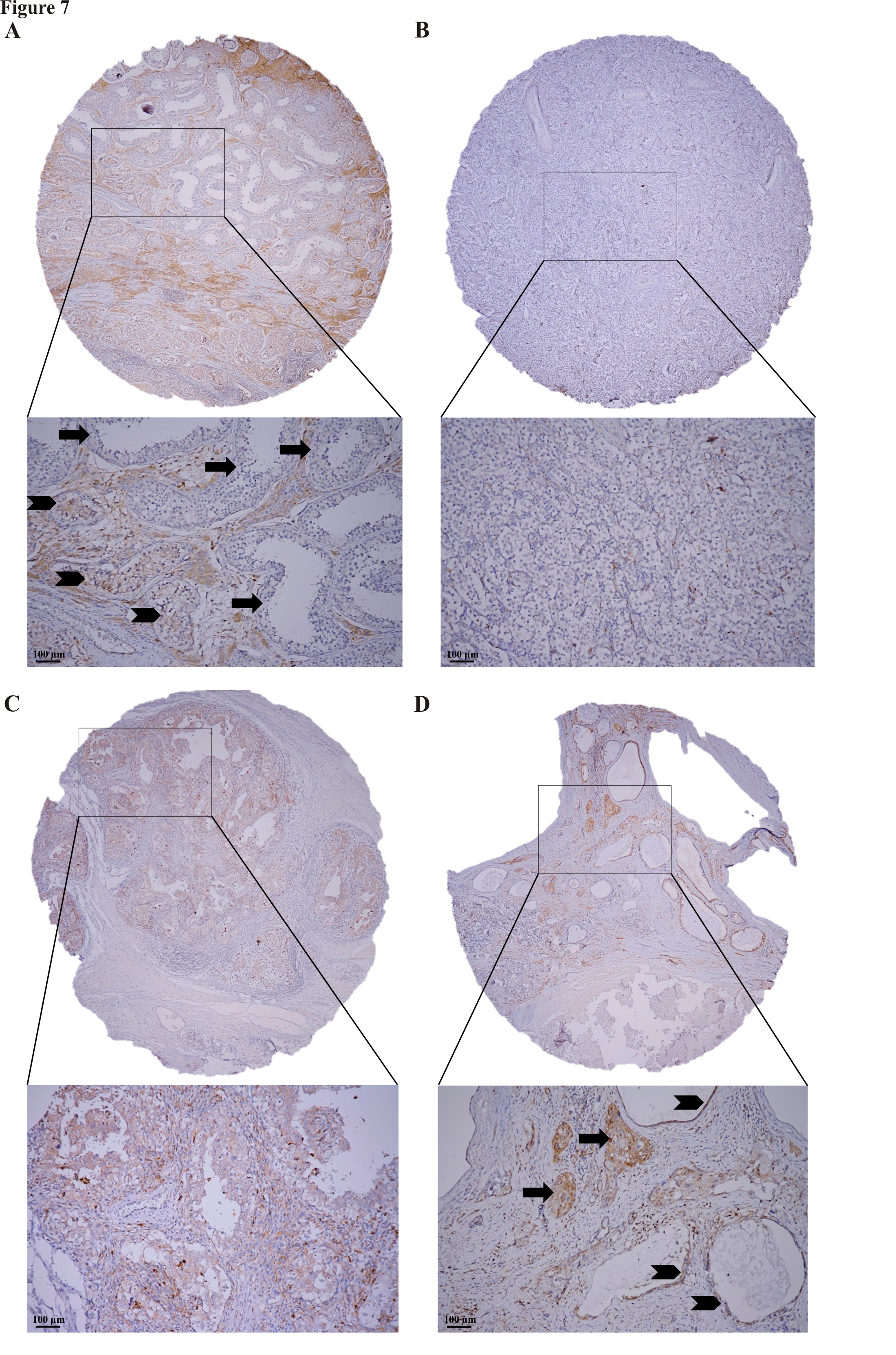

Supplement: Supplementary file 1 [file cancers-11-01224-s001.zip › fig 7.tif]

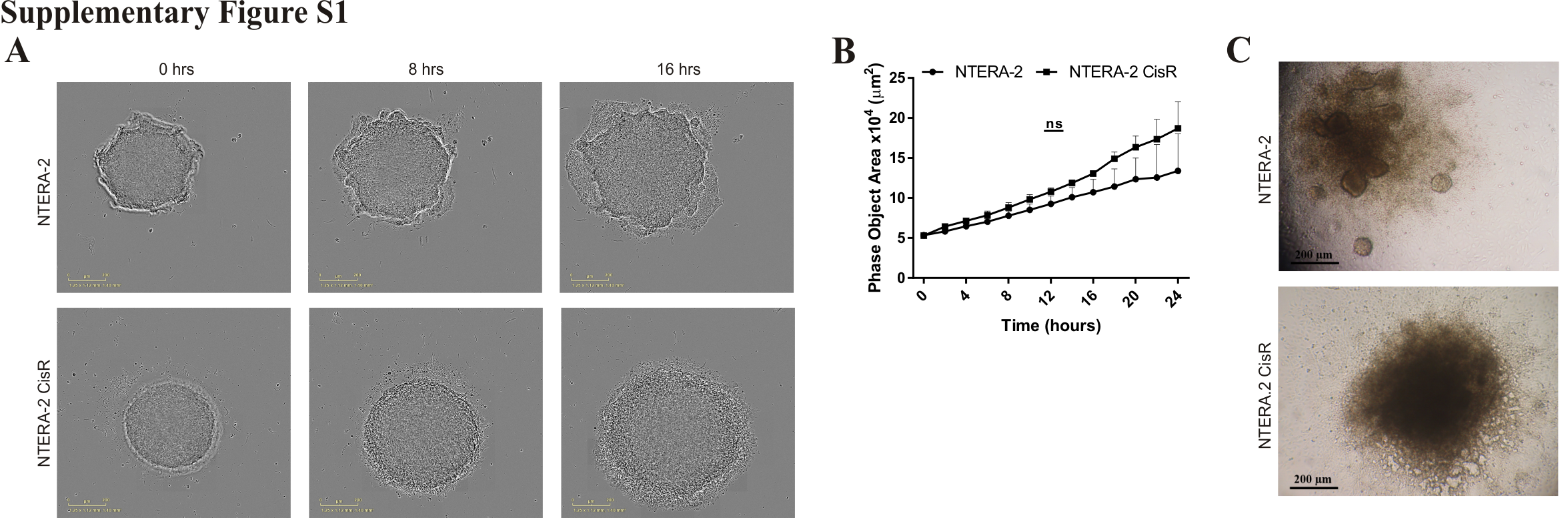

Supplement: Supplementary file 1 [file cancers-11-01224-s001.zip › Supp Fig S1.tif]

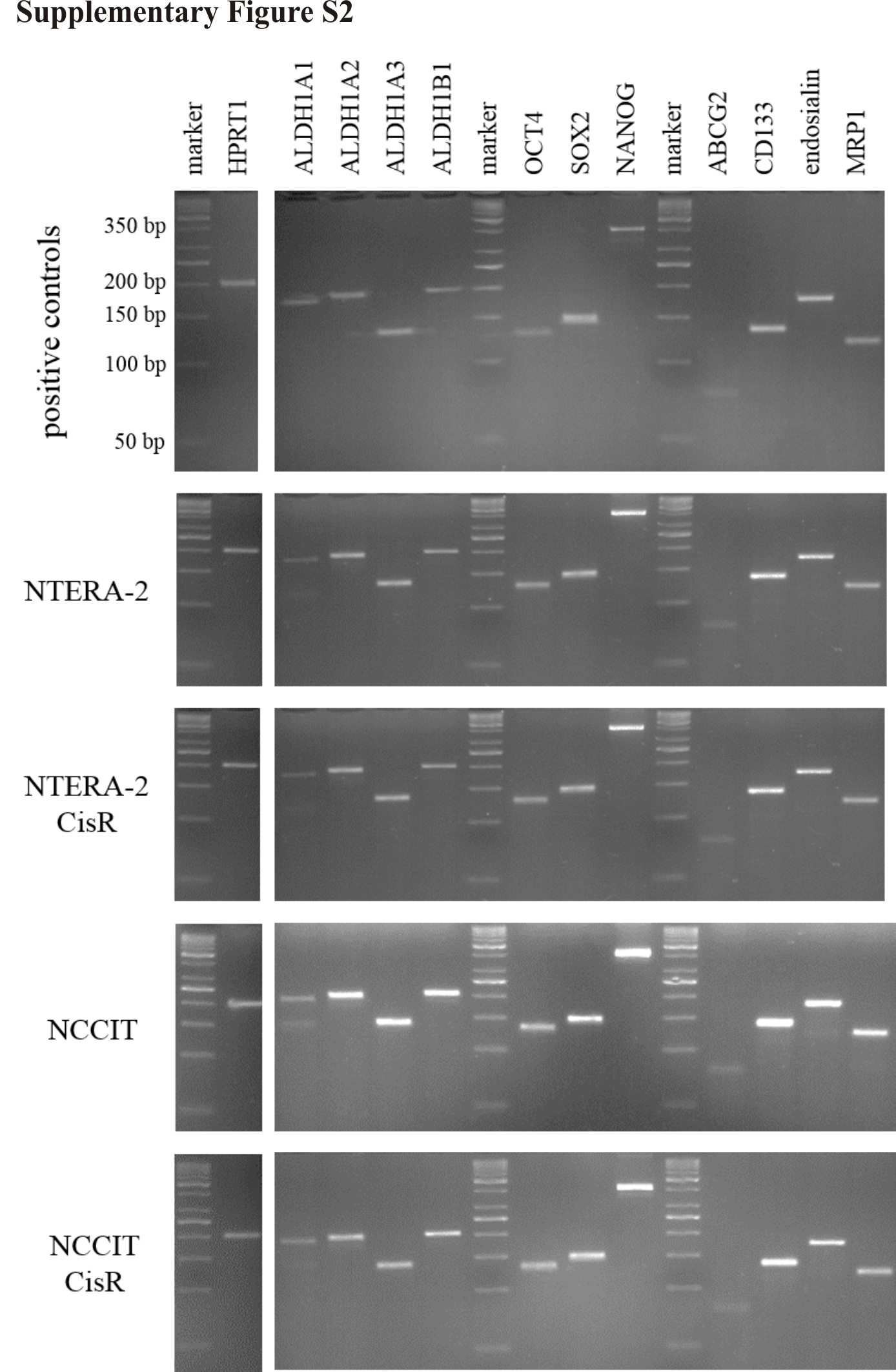

Supplement: Supplementary file 1 [file cancers-11-01224-s001.zip › Supp Fig S2.tif]

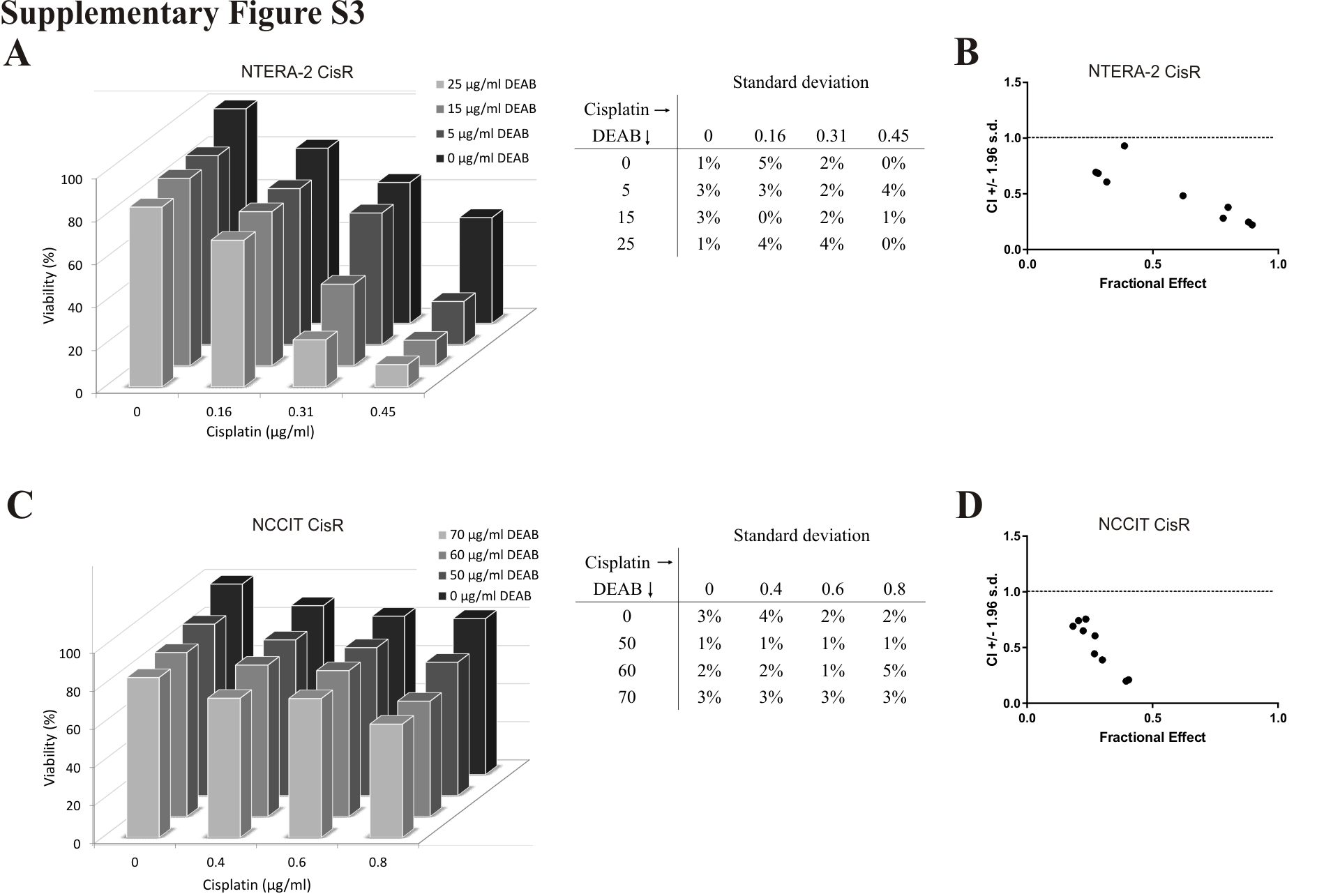

Supplement: Supplementary file 1 [file cancers-11-01224-s001.zip › Supp Fig S3.tif]
